# Functional Yogurt Enriched with *Moringa oleifera* Leaf Extract: Physicochemical, Sensory, Microbiological, and Antihyperglycemic Evaluation in Diabetic Rats

**DOI:** 10.3390/molecules31162764

**Published:** 2026-08-09

**Authors:** Beatriz Adriana Rodríguez-Romero, David Paniagua-Vega, Orlando Esau Flores-Maldonado, Aurora de Jesús Garza-Juárez, Ariana Arlene Huerta-Heredia, Perla Giovanna Silva-Flores

**Affiliations:** 1 Tecnológico de Monterrey, Escuela de Ingeniería y Ciencias, Monterrey 64849, Mexico; beatriz.rodriguez@tec.mx; 2 Facultad de Medicina, Universidad Autónoma de Nuevo León, Monterrey 64460, Mexico; david.paniaguav@uanl.edu.mx (D.P.-V.); orlando.floresmnd@uanl.edu.mx (O.E.F.-M.); aurora.garzajr@uanl.edu.mx (A.d.J.G.-J.); 3 Secretaría de Ciencia, Humanidades, Tecnología e Innovación (SECIHTI), Mexico City 03940, Mexico; ahuerta@unpa.edu.mx; 4 Instituto de Biotecnología, Centro de Investigaciones Científicas, Universidad del Papaloapan, San Juan Bautista Tuxtepec s/n, Oaxaca 68301, Mexico

**Keywords:** *Moringa oleifera*, functional yogurt, HPLC, DFI-ESI-IT-MS^2^, antihyperglycemic activity, Wistar rats

## Abstract

Functional foods enriched with plant-derived bioactive compounds have emerged as promising complementary dietary strategies to improve glycemic regulation. The aim of this study was to evaluate the physicochemical properties, sensory acceptance, and microbiological characteristics of a functional yogurt enriched with *Moringa oleifera* leaf extract, as well as its effect on blood glucose levels in an alloxan-induced diabetic rat model. Phytochemical characterization of the hydroethanolic leaf extract by HPLC and direct-infusion ESI-IT-MS^2^ revealed phenolic compounds, including chlorogenic acid, rutin, quercetin, and kaempferol, together with additional glycosylated flavonoids. *M. oleifera* extract increased the yogurt’s protein content. Sensory evaluation indicated moderate acceptance compared to natural yogurt, with color being the least-accepted attribute. Microbiological analysis showed no inhibitory effect against representative bacterial genera of the gastrointestinal microbiota under the experimental conditions evaluated. In vivo evaluation in alloxan-induced diabetic female Wistar rats demonstrated a significant reduction in blood glucose levels, from 396.50 ± 17.85 mg/dL to 254.50 ± 32.79 mg/dL after 15 days of treatment. These findings support further investigation of *M. oleifera* functional yogurt as a potential functional food in the context of glycemic control.

## 1. Introduction

Diabetes mellitus is a chronic metabolic disorder characterized by alterations in insulin secretion and/or insulin action, resulting in persistent hyperglycemia [1]. Prolonged elevated blood glucose levels are associated with severe complications, including cardiovascular disease, nephropathy, neuropathy, and retinopathy [1,2]. In Mexico, diabetes mellitus represents a major public health and socioeconomic challenge, as pharmacological treatment imposes a substantial financial burden on patients and healthcare systems [3]. Therefore, the development of complementary strategies that contribute to disease prevention and glycemic control has attracted increasing scientific interest [4].

In this context, functional foods have emerged as promising alternatives due to their potential to provide health benefits beyond basic nutrition. Functional foods may contain prebiotics, probiotics, or bioactive natural compounds that can exert beneficial physiological effects [5]. The Food and Agriculture Organization of the United Nations (FAO) defines functional foods as products that incorporate or enhance ingredients with potential health-promoting properties [6]. Yogurt is a widely consumed fermented dairy product produced by fermenting milk with lactic acid bacteria [7]. Yogurt consumption has been associated with multiple health benefits, including improved digestion and modulation of the intestinal microflora attributed to its probiotic content [8].

Plant-based bioactive compounds are an important source of functional ingredients due to their properties. In this regard, plant-based yogurt has emerged as a promising functional food with potential applications as a complementary dietary approach for metabolic disorders, including diabetes mellitus [9]. *Moringa oleifera*, a medicinal plant in the family Moringaceae, has attracted significant scientific attention due to its wide range of biological properties [10]. The leaves of *M. oleifera* are the most studied part of the plant and are recognized for their high nutritional value and abundance of bioactive compounds, including quercetin, catechin, kaempferol, chlorogenic acid, rutin, and various alkaloids [11,12]. These phytochemicals have been associated with antioxidant, antimicrobial, anti-inflammatory, antidiabetic, hepatoprotective, and anticancer activities [13]. Several studies have reported the antidiabetic potential of *M. oleifera* leaf extracts, showing their ability to reduce hyperglycemia and improve metabolic parameters in experimental models [14,15].

Animal models remain essential for evaluating the metabolic effects of functional foods under controlled experimental conditions. Wistar rats are widely used in metabolic research due to their well-characterized physiology and reproducible response to diabetogenic agents [16]. Alloxan-induced diabetes is a well-established experimental model characterized by selective pancreatic β-cell destruction mediated by oxidative stress, resulting in sustained hyperglycemia [17]. This model provides a reliable platform for assessing the antihyperglycemic potential of bioactive food formulations.

Although interest in plant-enriched functional dairy products has increased, limited information is available on incorporating *M. oleifera* extracts into yogurt formulations, particularly regarding their in vivo antihyperglycemic potential. Furthermore, most studies are restricted to compositional or nutritional evaluations, which limits their applicability to functional food development.

Therefore, the aim of this pilot study was to develop a functional yogurt with a hydroethanolic extract of *M. oleifera* leaves and to evaluate its physicochemical and sensory characteristics, in vitro microbiological activity, and its effect on blood glucose levels in an alloxan-induced diabetic rat model.

## 2. Results

### 2.1. Phenolic Profile of Moringa oleifera Leaf Extract by HPLC and DFI-ESI-IT-MS^2^

**Figure 1:**
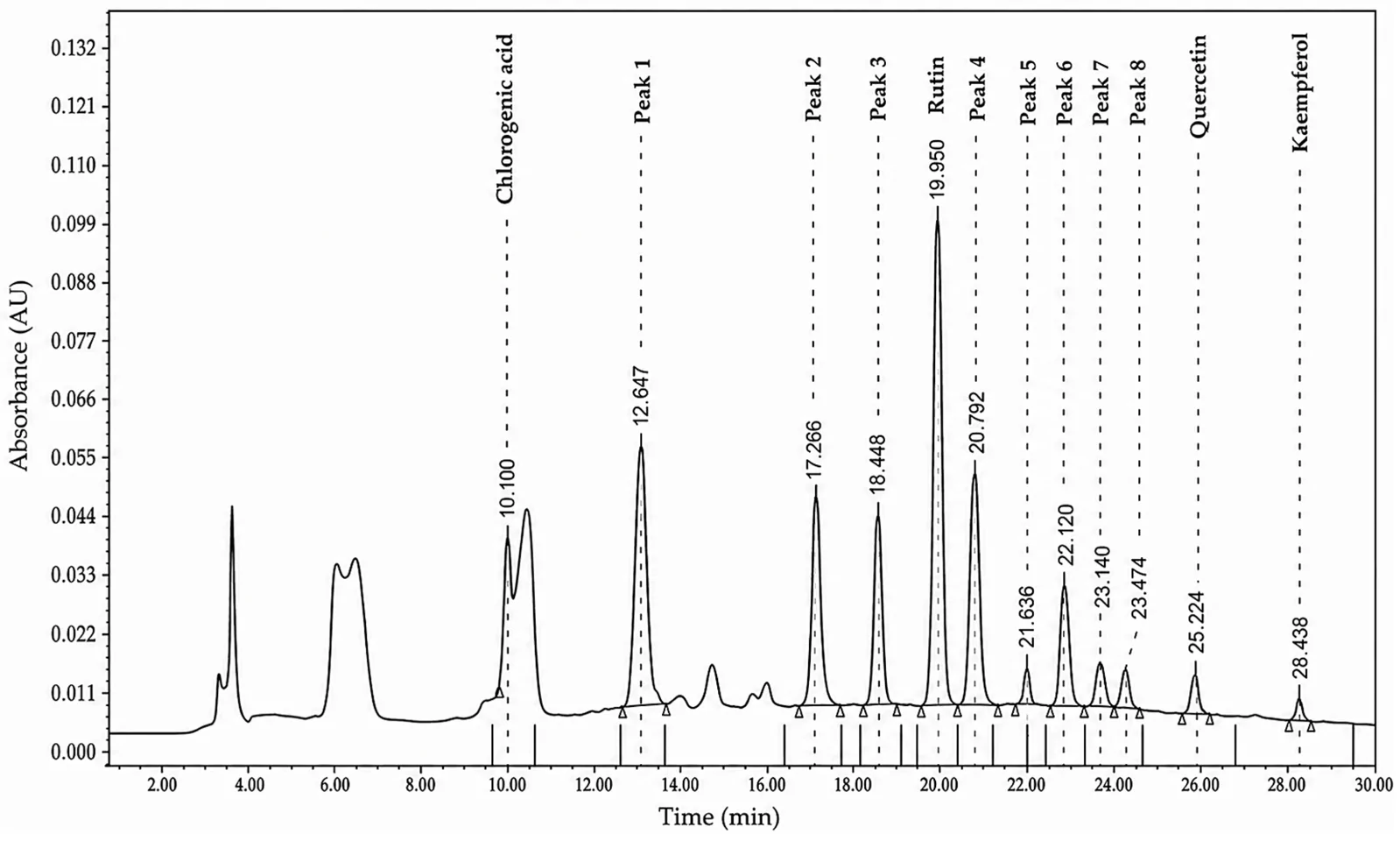
HPLC chromatogram of *Moringa oleifera* hydroethanolic extract. The dotted lines indicate retention times, while the solid lines below the peaks and the triangles indicate the integration ranges and limits, respectively.

The HPLC chromatographic profile of the *M. oleifera* leaf extract revealed four major phenolic compounds, including one phenolic acid (chlorogenic acid) and three flavonoids (rutin, quercetin, and kaempferol) (Figure 1). The assignment of these major compounds was further supported by direct-infusion ESI-IT-MS^2^ fragmentation analysis (Supplementary Figures S1–S3 and S5). Eight additional chromatographic peaks were observed in the HPLC profile. Complementary DFI-ESI-IT-MS^2^ analysis revealed ions tentatively assigned to myricetin, quinic acid, caffeic acid, kaempferol-O-hexoside, quercetin-O-hexoside, quercetin-O-acetyl-hexose, and apigenin-C-hexoside based on their characteristic fragmentation patterns (Supplementary Figures S4 and S6–S11).

### 2.2. Physicochemical Analysis of Yogurt

The physicochemical composition of natural and *Moringa* yogurt is presented in Table 1. Significant differences (*p* ≤ 0.05) in protein and pH values (day 0 and day 15) in both formulations were observed.

### 2.3. Sensory Evaluation of Yogurt

The sensory evaluation results of natural yogurt and *Moringa* yogurt are shown in Table 2. Significant differences (*p* ≤ 0.05) were observed except for the mouthfeel test.

### 2.4. In Vitro Microbiological Analysis

The microbial safety results for *M. oleifera* extract, natural yogurt, and *Moringa* yogurt are presented in Table 3. *M. oleifera* extract and natural yogurt did not contain total coliforms, yeasts, or molds. In contrast, *Moringa* yogurt presented low levels of total coliforms (2 ± 1 CFU/mL) and yeasts (1 ± 1 CFU/mL).

**Figure 2:**
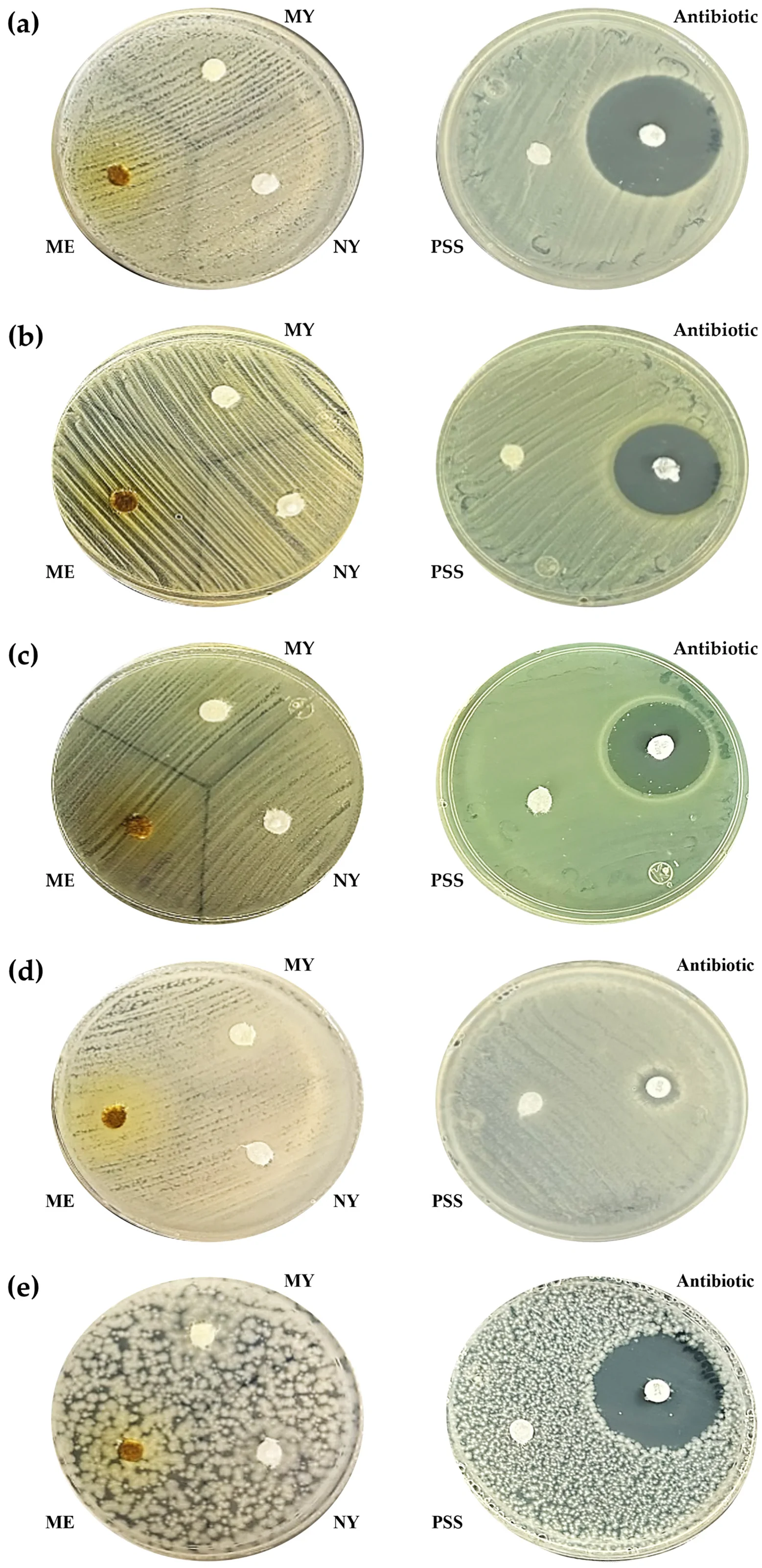
Antibacterial activity of natural yogurt (NY), *Moringa* yogurt (MY), and *M. oleifera* extract (ME), against (**a**) *Escherichia coli*, (**b**) *Staphylococcus aureus*, (**c**) *Pseudomonas aeruginosa*, (**d**) *Lactobacillus rhamnosus*, and (**e**) *Bifidobacterium lactis*, compared to antibiotic control and physiological saline solution (PSS).

The antibacterial activity of *M. oleifera* extract and *Moringa* yogurt was evaluated against Gram-positive and Gram-negative bacteria. The results showed that neither the hydroethanolic extract nor functional yogurt exhibited antibacterial activity against *E. coli*, *S. aureus*, *P. aeruginosa*, *L. rhamnosus,* and *B. lactis* (Figure 2).

### 2.5. In Vivo Antihyperglycemic Evaluation

**Figure 3:**
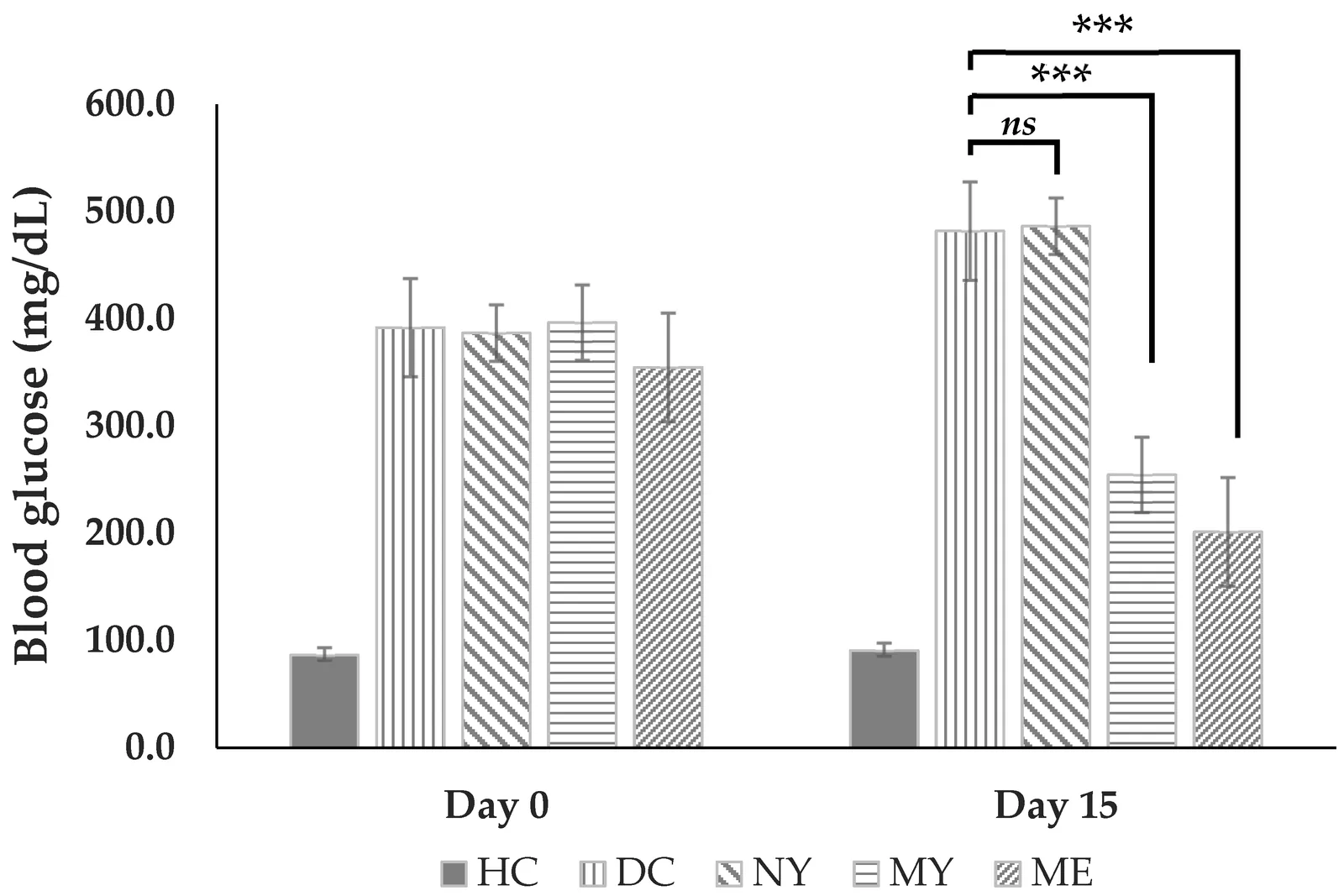
Blood glucose levels of the healthy control group (HC), diabetic group (DC), natural yogurt (NY), *Moringa* yogurt (MY), and *M. oleifera* extract (ME) (mean ± SD, n = 6). Statistical differences were analyzed using ANOVA with appropriate post hoc tests. *** Indicates significant difference (*p* < 0.001) compared to the diabetic control group; *ns*: not significant.

The average body weights of rats in the healthy control group (HC), diabetic control group (DC), natural yogurt group (NY), *Moringa* yogurt group (MY), and *M. oleifera* extract (ME) on days 1 and 15 of the study are presented in Table 4, while the corresponding blood glucose levels are shown in Figure 3. The repeated-measures ANOVA revealed a significant time × group interaction for blood glucose levels (F (4, 25) = 161.8, *p* < 0.001, partial η^2^ = 0.963), indicating that changes in blood glucose over time differed among the experimental groups. Analysis of change scores (day 15 − day 0) also showed significant differences among groups (Welch’s F (4, 10.3) = 239, *p* < 0.001). Games–Howell post hoc comparisons showed greater reductions in blood glucose in the MY and ME groups compared with the DC group (both *p* < 0.001), whereas the NY group did not differ significantly from the DC group (*p* = 0.787).

## 3. Discussion

### 3.1. Phenolic Profile of Moringa oleifera Leaf Extract by HPLC and DFI-ESI-IT-MS^2^

Detailed phytochemical characterization of the hydroethanolic *M. oleifera* leaf extract by HPLC and DFI-ESI-IT-MS^2^ revealed the presence of phenolic compounds commonly reported in the literature, including the major phenolic compounds chlorogenic acid, rutin, quercetin, and kaempferol, together with ions tentatively assigned to quinic acid and caffeic acid. The assignment of the major phenolic compounds was supported by comparison of chromatographic retention times with authentic standards, while DFI-ESI-IT-MS^2^ provided complementary structural information through characteristic fragmentation patterns. The observed precursor ions and MS^2^ fragmentation patterns were consistent with those obtained for authentic standards, when available, and with fragmentation patterns previously reported in the literature for chlorogenic acid, rutin, quercetin, kaempferol, and related phenolic constituents, thereby increasing confidence in compound assignment. Representative fragmentation spectra are provided in the Supplementary Material.

The phenolic profile identified in the *M. oleifera* leaf extract is consistent with previous chromatographic and mass spectrometric studies reporting phenolic acids and flavonoids, including quercetin, kaempferol, chlorogenic acid, and rutin [18,19]. These compounds identified in the *M. oleifera* leaf extract have been associated with antioxidant and antidiabetic properties [20,21]. However, because the phenolic composition was not characterized after incorporation of the extract into the yogurt matrix, a direct relationship between individual phenolic compounds and the reduction in blood glucose observed cannot be established based on the present findings.

### 3.2. Physicochemical Analysis of Yogurt

The extract concentration used to prepare the *Moringa* yogurt (5 g/L, equivalent to 0.5% *w*/*v*) was selected based on previous reports on *Moringa*-fortified dairy products, in which concentrations up to 0.5% were shown to preserve acceptable physicochemical and sensory properties [22]. The incorporation of *M. oleifera* extract increased the protein content on a dry-matter basis from 17.0 ± 1.53% in natural yogurt to 24.9 ± 2.14% in *Moringa* yogurt. This finding is consistent with previous reports by Karki et al., indicating increased protein content in dairy products supplemented with *M. oleifera* [23].

Titratable acidity, expressed as lactic acid, was comparable between *M. oleifera* yogurt (0.90 ± 0.48%) and natural yogurt (0.84 ± 0.15%), with values within previously reported ranges for yogurt products (0.72–0.97% and 0.69–1.81%) [24,25]. Thus, the incorporation of *M. oleifera* extract did not markedly affect this parameter.

Regarding acidity, both formulations showed a slight decrease in pH during storage, from 4.31 to 4.12 for natural yogurt and 4.45 to 4.21 for *Moringa* yogurt. This behavior is characteristic of fermented dairy products and is associated with the continued metabolic activity of lactic acid bacteria, which promotes the gradual production of organic acids. The values were consistent with those reported by Matela et al. [24], and remained below the 4.6 limit established by the U.S. Food and Drug Administration (FDA) for yogurt [26]. This value corresponds to the isoelectric point of casein and represents a critical parameter for determining the endpoint of milk fermentation. Furthermore, maintaining a pH below 4.6 helps inhibit the growth of pathogenic microorganisms, thereby improving product microbiological safety [24,25].

Overall, the incorporation of *M. oleifera* extract increased protein content without markedly altering the acidity and pH characteristics of the yogurt, supporting its feasibility as an ingredient in the evaluated formulation.

### 3.3. Sensory Evaluation of Yogurt

Natural yogurt received the highest approval rating, as evaluators noted its organoleptic characteristics. For the functional yogurt, the sensation when swallowing obtained the highest approval rating, whereas the smell and taste attributes showed moderate acceptance. Color received the lowest approval rating and exhibited the most pronounced difference between formulations (*p* < 0.001). Overall acceptance of the fortified yogurt ranged from moderate to slightly unpleasant, identifying color as the main sensory attribute requiring improvement. Therefore, reformulation should primarily address this attribute to improve hedonic acceptance and future product development. This sensory evaluation was designed as an exploratory acceptance test for a newly developed prototype to assess overall liking and identify potential barriers to consumer acceptance, rather than as a confirmatory consumer validation or product optimization study [27]. The panel size used in this study (*n*= 30) is consistent with methodological recommendations for exploratory acceptance tests when the objective is overall acceptance rather than consumer segmentation [27,28], and is comparable to that used in previous sensory studies on *Moringa*-enriched yogurt (e.g., Wang et al. *n*= 10 trained tasters, and Kuikman and O’Connor *n*= 37 untrained consumers [25,29]). Consequently, demographic characterization, consumer segmentation, and sensory evaluation after prolonged storage fall outside the exploratory scope defined for this pilot study. However, consumer acceptance remains a key factor in the successful development of functional foods, and the product’s color poses a significant barrier that should be addressed through reformulation in future studies.

### 3.4. In Vitro Microbiological Analysis

The microbiological criteria established to ensure yogurt safety are defined in accordance with the applicable regulatory framework. According to the Official Mexican Standard (NOM-243-SSA1-2010), fermented dairy products must comply with limits for indicator microorganisms, such as total coliforms, yeasts, and molds, as well as minimum viable counts of lactic acid bacteria to ensure product quality and safety [30]. Similarly, European Commission Regulation (EC) No 2073/2005 establishes microbiological criteria based on specific target microorganisms and food categories [31]. Within this regulatory context, the microbiological results were consistent with the applicable microbiological criteria evaluated in this study.

Furthermore, neither the hydroethanolic extract nor the functional yogurt exhibited detectable antibacterial activity against the bacterial strains evaluated under the experimental conditions employed. Previous studies have likewise reported that yogurt fortified with *M. oleifera* leaf extract, which was developed as a nutritional strategy to mitigate child malnutrition, did not exhibit antibacterial activity against *E. coli* or *S. aureus* [32]. However, this study focused primarily on product formulation and did not evaluate biological effects on metabolic parameters.

### 3.5. In Vivo Antihyperglycemic Evaluation

The diabetic model employed in the present study was induced using alloxan, a diabetogenic agent that selectively damages pancreatic β-cells and is commonly associated with experimental models of type 1 diabetes. Nevertheless, alloxan-induced hyperglycemia remains one of the most widely used and reproducible experimental models for the preliminary screening of glucose-lowering effects of natural products and plant-derived compounds [33]. The inclusion of a healthy control group also facilitated the interpretation of treatment-related effects by allowing comparisons with normal physiological status. The healthy control group (HC) remained normoglycemic and exhibited no marked changes in body weight throughout the experimental period, maintaining blood glucose levels from 86.33 ± 4.80 mg/dL at baseline to 90.67 ± 6.25 mg/dL on day 15 of the study (Figure 3). These findings provide a baseline reference for normal physiological conditions. In contrast, diabetic animals displayed the characteristic alterations associated with alloxan-induced hyperglycemia, confirming the successful establishment of the diabetic model. Accordingly, the untreated diabetic control group (DC) showed an increase in mean blood glucose levels, from 391.83 ± 33.28 mg/dL to 481.83 ± 38.14 mg/dL on day 15. A similar trend was observed in the experimental group receiving natural yogurt, with glucose levels increasing from 386.83 ± 23.52 mg/dL to 486.33 ± 24.53 mg/dL. In contrast, the experimental group treated with the hydroethanolic extract of *M. oleifera* showed a marked reduction in mean glucose values, decreasing from 354.67 ± 64.34 mg/dL to 201.33 ± 45.36 mg/dL. This glucose-lowering response is consistent with that reported by Abd et al. in diabetic rats treated with a crude extract of *M. oleifera*, in which mean blood glucose levels decreased from approximately 400 mg/dL to 200 mg/dL, supporting the glucose-lowering potential of the extract [34]. Likewise, the group receiving *Moringa* yogurt exhibited a decrease in mean glucose levels from 396.50 ± 17.85 mg/dL to 254.50 ± 32.79 mg/dL on day 15 of the pilot study. The daily dose of 2 mL corresponds to an estimated intake of approximately 10 mg of extract per rat per day (≈43 mg/kg of body weight), calculated based on the average initial body weight of the group that consumed *Moringa* yogurt (Table 4). Although this dose is lower than that used in studies in which isolated *M. oleifera* extract is administered (100–800 mg/kg), this value is comparable to the doses that have been reported to produce the antidiabetic effect of *M. oleifera* as part of a food matrix, rather than as a concentrated extract [35,36].

Previous in vitro and in vivo studies have reported glucose-lowering effects of *M. oleifera* leaf extracts through mechanisms including inhibition of key carbohydrate-digesting enzymes (α-amylase and α-glucosidase) and modulation of oxidative stress and glucose metabolism [37,38,39,40]. For instance, rutin has been reported to reduce carbohydrate absorption from the small intestine and improve antioxidant status in diabetic rats [41,42]. Quercetin has been shown to enhance insulin secretion and protect pancreatic β-cells under diabetic conditions [43], while more recent evidence supports its ability to inhibit the enzymes α-amylase and α-glucosidase [44]. Similarly, chlorogenic acid has been associated with α-glucosidase inhibition and improved insulin resistance in HepG2 cells [45]. These previous findings provide a plausible biological context for the reduction in blood glucose observed in the present study. However, these mechanisms were not investigated and therefore cannot be established based on the present findings.

A reduction in body weight was observed in diabetic animals during the experimental period. This finding is consistent with the metabolic alterations associated with alloxan-induced diabetes, including impaired glucose utilization, enhanced proteolysis, and increased lipid mobilization, which commonly result in body weight loss in diabetic animals [17,33]. The fact that this response was observed across all diabetic groups suggests that the reduction in body weight was primarily associated with the diabetic condition rather than with the administered treatments. Furthermore, previous studies have reported the safety of *M. oleifera* at comparable doses, with no evidence of treatment-related toxicity in experimental models [46,47]. Nevertheless, the potential contribution of the yogurt matrix to nutrient intake and metabolic responses cannot be entirely ruled out and warrants further investigation.

To the best of our knowledge, no previous studies have specifically evaluated a functional yogurt containing *Moringa oleifera* leaf extract together with its effect on blood glucose levels in an experimental model. Therefore, the present pilot study provides additional evidence for the feasibility of incorporating *M. oleifera* into a functional dairy product and lays the groundwork for future investigations. Because the effect on blood glucose levels was evaluated using an alloxan-induced hyperglycemic model, the findings should be interpreted within the limitations of this experimental model, which does not fully reproduce the complex pathophysiology of other types of diabetes.

## 4. Materials and Methods

### 4.1. Materials

*Moringa oleifera* leaves were obtained from plants grown from seeds germinated in Peat Moss^®^, achieving a germination rate of 87.8 ± 4.3%. Seedlings were subsequently cultivated in a greenhouse under protected conditions using drip irrigation and nutrient supplementation [18]. Whole cow’s milk was purchased from commercial stores. Starter culture of lactic acid bacteria *Streptococcus salivarius* subsp. *thermophilus* and *Lactobacillus delbrueckii* subsp. *bulgaricus* was obtained from LyoPro^®^ and CodeXing Biotech Ingredients^®^ (Miami, FL, USA) [48]. HPLC-grade methanol was obtained from Fermont^®^, México. Alloxan monohydrate and the rest of the reagents were purchased from Sigma-Aldrich^®^ (St. Louis, MO, USA). Standard agar, brilliant red-green bile agar, Sabouraud agar with chloramphenicol, and malt agar were obtained from BD (Bioxon^®^, Mexico City, Mexico).

### 4.2. Moringa oleifera Extract

Fresh leaves were air-dried and ground into powder. Hydroethanolic extract of *M. oleifera* (ME) leaves was obtained by mixing the leaf powder into 80% ethanol (1:10 *v*/*v*) for 20 min with orbital shaking at 150 rpm (Unimax 1010, Heidolph Instruments^®^ GmbH & CO. KG, Schwabach, Germany) [18]. Subsequently, the volume of three successive extractions was concentrated under reduced pressure at 40 °C (Hei-VAP Core, Heidolph Instruments GmbH & Co. KG, Schwabach, Germany). The extraction process resulted in a yield of 29.2 ± 2.0% (*w*/*w*, dry basis). The concentrate was freeze-dried and stored at −4 °C until use.

### 4.3. Phenolic Profile of Moringa oleifera Leaf Extract by HPLC and DFI-ESI-IT-MS^2^

HPLC analysis was performed using a Waters modular liquid chromatography system (Waters^®^, Milford, MA, USA) equipped with a 1525 binary pump, an in-line degasser, a 717 Plus autosampler, and a 2996 diode-array detector (DAD). Data acquisition and processing were carried out using Empower 3 software (Waters^®^, Milford, MA, USA).

Chromatographic separation was achieved on a Luna RP-C18 column (150 × 4.6 mm, 5 µm), fitted with a Luna C18 guard column (4 × 3 mm, 5 µm) (Phenomenex^®^, Torrance, CA, USA). The injection volume was 20 µL of hydroethanolic extract of *M. oleifera* leaf, previously diluted in 80% methanol and filtered through a 0.45 µm membrane (Durapore^®^, Millipore, Ireland). The flow rate was set at 0.5 mL min^−1^. The mobile phase consisted of methanol (A) and 0.1% formic acid in water (B), using the following gradient program: 70% B at 0 min, decreasing to 40% B over 0–15 min, maintained at 40% B for 15–20 min, and further reduced to 30% B over 20–30 min. Detection was performed at 350 nm.

Major phenolic compounds were initially identified by comparison of their chromatographic retention times and UV spectra with those of authentic reference standards. Compound assignment was further supported by direct-infusion electrospray ionization ion-trap tandem mass spectrometry (DFI-ESI-IT-MS^2^). For DFI-ESI-IT-MS^2^ analysis, the hydroethanolic extract and authentic reference standards were analyzed in the negative ion mode. Precursor ions were subjected to collision-induced dissociation (CID), and compound assignment was based on comparison of the resulting MS^2^ fragmentation patterns with those of authentic standards, when available, and with fragmentation data previously reported in the literature and available in spectral databases, including MassBank of North America (MoNA), NORMAN MassBank, and PhytoHub [49,50,51]. Representative precursor ion and MS^2^ fragmentation spectra are provided in the Supplementary Material.

### 4.4. Yogurt Preparation

Yogurt was prepared according to the protocol described by Nole-Jaramillo et al. [52] with minor modifications. *Moringa* yogurt (MY) was produced by adding 100 g of sucrose to 1000 mL of pasteurized whole cow’s milk at 43–45 °C.

Subsequently, 100 mg of the starter culture inoculum (*Lactobacillus delbrueckii* subsp. *bulgaricus* and *Streptococcus thermophilus*) was dissolved in 100 mL of the milk-sugar mixture. After complete dissolution, the inoculated portion was added back to the remaining milk and homogenized to obtain a final concentration of approximately 108 CFU/mL.

The mixture was incubated at 42 °C for approximately 4 h until the pH reached 4.3. After fermentation, 5 g of hydroethanolic *M. oleifera* extract (equivalent to 5 g/L) was added to the yogurt [53]. The mixture was homogenized and stored at 6 ± 2 °C until physicochemical and sensory analyses were performed.

Natural yogurt (NY), prepared without *M. oleifera* extract, was produced under the same conditions. Three independent batches were prepared for each formulation.

### 4.5. Physicochemical Analysis

The physicochemical characterization of yogurt included the determination of pH, titratable acidity, protein content, and total sugars. pH and titratable acidity were determined according to the methodology described by Nole-Jaramillo et al. [52]. The electrode was directly immersed in 25 mL of the yogurt sample until a stable reading was obtained at Day 0 and Day 15. Titratable acidity was determined by titrating a 10% (*w*/*v*) diluted yogurt sample, mixed with phenolphthalein indicator, against 0.1 N sodium hydroxide solution. Results were expressed as a percentage of lactic acid (%). Analyses were performed at room temperature unless otherwise specified.

Protein contents were determined according to the methods established by the Association of Official Analytical Chemists (AOAC) and Mexican standards [54,55,56]. All analyses were performed in triplicate.

### 4.6. Sensory Evaluation

The sensory properties of yogurt were evaluated following the methodology described by Campos et al. [57] with minor modifications. Sensory analysis was conducted after 1 day of refrigerated storage. A total of 30 untrained panelists from the Faculty of Agronomy, UANL, Mexico, participated in the evaluation. Sensory tests were performed in a laboratory designed in accordance with ISO 8589:2007 [58].

Yogurt samples (natural yogurt and *Moringa* yogurt) were served simultaneously. Approximately 15 mL of each sample was placed in individual plastic cups coded with random three-digit numbers.

The sensory attributes evaluated were color, aroma, flavor, mouthfeel, and overall acceptability. A five-point hedonic scale was used, where (1) very dissatisfied, (2) dissatisfied, (3) neither like nor dislike, (4) like, and (5) like very much. Before evaluation, participants were informed about the study objectives and voluntarily agreed to participate in the tasting session.

### 4.7. In Vitro Microbiological Analysis

#### 4.7.1. Microbial Safety

Total coliforms, yeasts, and molds were determined using Brilliant Green Bile Agar, Sabouraud agar supplemented with chloramphenicol, and Malt agar, respectively, following the Mexican Standard (NOM-243-SSA1-2010) and internationally recognized microbiological methods [30,59,60]. Briefly, the extract and yogurt solutions were prepared by serial dilution in saline solution (1:10), and 100 µL of each solution was then inoculated into the culture media. Brilliant Green Bile Agar plates were incubated at 37 °C for 24 h, while the Sabouraud agar with chloramphenicol and Malta agar plates were incubated at 25 °C for 7 days. After the incubation period, the colony-forming units per mL (CFU/mL) were counted.

#### 4.7.2. Antibacterial Activity

Antibacterial activity of *M. oleifera* extract, natural yogurt, and *Moringa* yogurt was evaluated using the agar disc diffusion assay following standardized antimicrobial susceptibility testing protocols [61]. Five bacterial strain suspensions were evaluated: (a) *Escherichia coli*, (b) *Staphylococcus aureus*, (c) *Pseudomonas aeruginosa*, (d) *Lactobacillus rhamnosus*, and (e) *Bifidobacterium lactis*. Bacterial suspensions were adjusted to an optical density of 0.1 at 600 nm (OD600). A standardized bacterial inoculum was uniformly spread on sterile agar plates using a sterile cotton swab. Subsequently, sterile filter paper discs were placed onto the agar surface. Each disc was inoculated with 7 µL of one of the following treatments: (i) *M. oleifera* extract (0.5 g/mL) (ME), (ii) natural yogurt (NY), or (iii) *Moringa* yogurt (MY). The inoculated plates were incubated at 37 °C for 24 h under aerobic conditions. After incubation, antibacterial activity was determined by the presence of inhibition zones (halos). All treatments were evaluated in triplicate.

### 4.8. In Vivo Antihyperglycemic Evaluation

#### 4.8.1. Ethical Approval of Animal Use

The use of experimental animals was approved by the Institutional Animal Care and Use Committee of the Faculty of Medicine, UANL (protocol number FI25-00005). All experimental procedures were conducted in accordance with the provisions of the Official Mexican Standard (NOM-062-ZOO-1999) [62] and were consistent with the Guide for the Care and Use of Laboratory Animals published by the National Research Council [63]. Animals were monitored daily for general health status, signs of distress, and adverse effects throughout the experimental period. All procedures were performed under the strict supervision of a certified veterinarian.

#### 4.8.2. Experimental Design and Animals

Thirty (30) healthy female albino Wistar rats weighing 250 ± 50 g were used for in vivo experiments. The animals were housed in polypropylene cages under standard housing conditions (temperature range of 25 ± 2 °C, 12 h light/12 dark cycle, and 35–60% relative humidity). Animals were fed *ad libitum* with standard commercial rat food (Formulab Diet 5008, Nutrimix^®^, México City, México) and had free access to filtered drinking water. The sample size was determined by an a priori power analysis performed using G*Power software to ensure adequate statistical power while adhering to the principles of Reduction, Refinement, and Replacement (3 Rs) in animal experimentation.

Following hyperglycemia induction, animals exhibiting ≥200 mg/dL were randomly allocated to four experimental groups (n = 6 per group): diabetic control (DC), natural yogurt (NY), *Moringa* yogurt (MY), and *M. oleifera* extract group (ME). The healthy control group (HC) (n = 6 rats) did not receive alloxan or any experimental treatment and was maintained concurrently under the same husbandry conditions as the other groups. Each animal was considered an independent experimental unit for all analyses.

#### 4.8.3. Experimental Phase

Diabetes mellitus was induced in rats by a single intraperitoneal injection of alloxan (150 mg/kg body weight) after an overnight fasting period [33,64]. In the present study, the antihyperglycemic effect was evaluated based on the ability of the intervention to reduce elevated blood glucose levels during the 15-day experimental period. Blood glucose levels were measured 72 h after alloxan administration by a certified veterinarian who was blinded to group allocation throughout the experimental period. The second blood drop was collected via tail-vein puncture, and glucose levels were determined using a Contour Plus^®^ glucometer. Wistar rats that met the criteria for hyperglycemia were included in the experimental groups. Treatments (natural yogurt, *Moringa* yogurt, and *M. oleifera* extract) were administered via orogastric gavage using a 16-gauge stainless-steel feeding cannula to ensure a controlled intake of 2 mL of each sample. Based on the *M. oleifera* extract concentration used to prepare the *Moringa* yogurt (5 g/L), the 2 mL daily dose corresponded to an estimated intake of approximately 10 mg of extract per rat per day. Treatments were administered daily, and blood glucose levels were monitored for 15 consecutive days.

### 4.9. Statistical Analysis

An a priori sample size calculation was performed using G*Power software (version 3.1) for a repeated-measures ANOVA design. Assuming a conservative large effect size (*f* = 0.60), a significance level of 0.05, a statistical power of 80%, five study groups, and two repeated measurements, the estimated minimum sample size was 30 animals (n = 6 per group).

Statistical analyses were performed using jamovi (version 2.6.44; The jamovi project, Sydney, Australia). Data were expressed as mean ± standard deviation (SD). Hedonic test results were analyzed using the nonparametric Friedman test, followed by Mood’s median test for pairwise comparisons. For the in vivo evaluation, a mixed repeated-measures ANOVA was conducted with time (day 0 and day 15) as the within-subject factor and group (HC, DC, NY, MY, and ME) as the between-subject factor. Partial eta squared (η^2^p) was reported as an effect size estimate for the repeated-measures ANOVA. Model assumptions were assessed prior to analysis: residual normality was evaluated using Q–Q plots, and homogeneity of variances using Levene’s test. Because the within-subject factor had only two levels, the assumption of sphericity was inherently satisfied. To further explore significant interactions, change scores (day 15 − day 0) were analyzed using one-way ANOVA. When homogeneity of variances was not met, Welch’s ANOVA followed by Games–Howell post hoc comparisons were applied. Statistical significance was set at *p* ≤ 0.05.

## 5. Conclusions

In this study, a functional yogurt enriched with a hydroethanolic extract of *Moringa oleifera* leaves was successfully developed and evaluated through physicochemical, microbiological, sensory, phytochemical, and biological evaluations. The developed yogurt complied with microbiological safety standards, showed acceptable technological properties, and exhibited moderate sensory acceptance, with color identified as the main factor limiting consumer acceptance. The encouraging physicochemical, microbiological, sensory, and preliminary antihyperglycemic results obtained in this study provide a basis for future investigations aimed at characterizing the final yogurt formulation, including the stability of its phenolic compounds during refrigerated storage and under larger-scale production conditions.

Phytochemical characterization of the *M. oleifera* leaf extract by HPLC and direct-infusion ESI-IT-MS^2^ fragmentation analysis supported the identification of phenolic compounds previously associated with antihyperglycemic activity, including chlorogenic acid, rutin, quercetin, and kaempferol. Although the contribution of individual compounds was not specifically investigated, the developed functional yogurt significantly reduced blood glucose levels in alloxan-induced diabetic rats, demonstrating a preliminary antihyperglycemic effect under the experimental conditions evaluated, while exhibiting no detectable antibacterial activity against the representative gastrointestinal bacterial genera evaluated.

To the best of our knowledge, this study is among the first to combine detailed phytochemical characterization of *M. oleifera* leaf extract with the development and in vivo evaluation of a functional yogurt formulation. These findings support the potential of *M. oleifera*-enriched yogurt as a functional food candidate for further investigation and provide a foundation for future studies evaluating its biological effects and potential applications.

## 6. Limitations

Although the present study provides novel evidence supporting the antihyperglycemic potential of a functional yogurt enriched with *M. oleifera* leaf extract, several limitations should be acknowledged. Although the phenolic profile of the *M. oleifera* leaf extract was comprehensively characterized by HPLC and direct-infusion ESI-IT-MS^2^, the contribution of individual compounds or their potential synergistic interactions to the observed antihyperglycemic activity was not specifically investigated. The biological evaluation was performed using an alloxan-induced diabetic rat model, which is widely accepted for the preliminary screening of antihyperglycemic activity but does not fully reproduce the complex pathophysiology of all forms of diabetes. Furthermore, the study focused on evaluating the biological activity of the developed functional yogurt and did not investigate the molecular mechanisms underlying the observed glucose-lowering effect. Another limitation of the present study is the absence of a pharmacological reference group (e.g., metformin), which would have allowed direct comparison between the functional yogurt and an established antihyperglycemic therapy. Future studies should evaluate dose–response relationships, elucidate the underlying molecular mechanisms, and validate these findings in additional preclinical models and well-designed clinical studies.

## Figures and Tables

**Table 1:** Physicochemical properties of yogurts (mean ± SD, *n* = 3).

Sample	Lactic Acid (%)	Protein in Dry Matter Basis (%)	pH (Day 0)	pH (Day 15)
Natural yogurt	0.84% ± 0.15	17.0% ± 1.53 ^a^	4.31 ± 0.04 ^a^	4.12 ± 0.03 ^a^
*Moringa* yogurt	0.90% ± 0.48	24.9% ± 2.14 ^b^	4.45 ± 0.05 ^b^	4.21 ± 0.05 ^b^

The results are expressed as means ± standard deviations of three independent measurements. ^a,b^ Different letters in the same column indicate significant differences (*p* ≤ 0.05).

**Table 2:** Average value of organoleptic evaluation using the hedonic test.

Attribute	Natural Yogurt	*Moringa* Yogurt	*p* ≤ 0.05
Color	4.00 ^a^	2.00 ^b^	0.000
Aroma	4.00 ^a^	3.00 ^b^	0.000
Flavor	4.00 ^a^	3.50 ^b^	0.018
Mouth feel	4.00	4.00	0.680
Overall acceptability	4.00 ^a^	3.00 ^b^	0.003

Five-point hedonic scale: (1) very dissatisfied, (2) dissatisfied, (3) neither like nor dislike, (4) like, and (5) like very much. The result values represent the median (*n* = 30). ^a,b^ Different letters in the same line indicate significant differences (*p* ≤ 0.05).

**Table 3:** Microbial analysis of *Moringa* extract, natural yogurt, and *Moringa* yogurt.

Sample	Coliforms	Yeast	Molds
*Moringa* extract	0 CFU/mL	0 CFU/mL	0 CFU/mL
Natural yogurt	0 CFU/mL	0 CFU/mL	0 CFU/mL
*Moringa* yogurt	2 ± 1 CFU/mL	1 ± 1 CFU/mL	0 CFU/mL

The results are expressed as means ± standard deviations of three independent measurements.

**Table 4:** Body weight (g) of rats on day 0 and day 15 of the study (mean ± SD, n = 6).

Group	Day 0	Day 15
Healthy control (HC)	248 ± 2.87	243 ± 3.09
Diabetic control (DC)	246 ± 2.59 ^a^	229 ± 3.19 ^b^
Natural yogurt (NY)	258 ± 2.76 ^a^	211 ± 5.23 ^b^
*Moringa* yogurt (MY)	233 ± 3.11 ^a^	198 ± 3.28 ^b^
*M. oleifera* extract (ME)	252 ± 1.53 ^a^	227 ± 1.45 ^b^

The results are expressed as means ± standard deviations. ^a,b^ Different letters in the same line indicate significant differences (*p* ≤ 0.05).

## Data Availability

The original contributions presented in this study are included in the article/supplementary material. Further inquiries can be directed to the corresponding author.

## Supplementary Materials

The following supporting information can be downloaded at: https://www.mdpi.com/article/10.3390/molecules31162764/s1, Supplementary Figure S1: Direct-infusion ESI-IT-MS^2^ analysis of chlorogenic acid; Figure S2: Direct-infusion ESI-IT-MS^2^ analysis of kaempferol; Figure S3: Direct-infusion ESI-IT-MS^2^ analysis of quercetin; Figure S4: Direct-infusion ESI-IT-MS^2^ analysis of myricetin; Figure S5: Direct-infusion ESI-IT-MS^2^ analysis of rutin; Figure S6: Direct-infusion ESI-IT-MS^2^ analysis of quinic acid; Figure S7: Direct-infusion ESI-IT-MS^2^ analysis of caffeic acid; Figure S8: Direct-infusion ESI-IT-MS^2^ analysis of kaempferol-O-hexoside; Figure S9: Direct-infusion ESI-IT-MS^2^ analysis of quercetin-O-hexoside; Figure S10: Direct-infusion ESI-IT-MS^2^ analysis of quercetin-O-acetyl-hexose; Figure S11: Direct-infusion ESI-IT-MS^2^ analysis of apigenin-C-hexoside.

## Abbreviations

The following abbreviations are used in this manuscript:

CFU	colony-forming units per mL
DC	Diabetic control group
DFI-ESI-IT-MS^2^	Direct-Infusion Electrospray Ionization Ion-Trap Tandem Mass Spectrometry
DM	Diabetes mellitus
HC	Healthy control group
HPLC	High-Performance Liquid Chromatography
ME	*Moringa oleifera* extract
MY	*Moringa* yogurt
NY	Natural yogurt
OD600	Optical Density at 600 nm
PSS	Physiological Saline Solution
SD	Standard Deviation
