# Supplementary material for: Functional Yogurt Enriched with Moringa oleifera Leaf Extract: Physicochemical, Sensory, Microbiological, and Antihyperglycemic Evaluation in Diabetic Rats
Source: Molecules. 2026 Aug 9;31(16):2764. doi: 10.3390/molecules31162764 (PMC13515957; doi:10.3390/molecules31162764)
Supplement: Supplementary file 1 [file molecules-31-02764-s001.zip › molecules-4443202-supplementary.pdf]

molecules

MDPI

*Supplementary Materials*

# Functional Yogurt Enriched with Moringa oleifera Leaf Extract: Physicochemical, Sensory, Microbiological, and Antihyperglycemic Evaluation in Diabetic Rats

**Beatriz Adriana Rodríguez-Romero [1,†], David Paniagua-Vega [2,3,†], Orlando Esau Flores-Maldonado [2], Aurora de Jesús Garza-Juárez [2], Ariana Arlene Huerta-Heredia [3,4] and Perla Giovanna Silva-Flores [2,*]**

[1] Tecnológico de Monterrey, Escuela de Ingeniería y Ciencias, Tecnológico de Monterrey, Monterrey 64849, Mexico; beatriz.rodriguez@tec.mx
[2] Universidad Autónoma de Nuevo León, Facultad de Medicina, Universidad Autónoma de Nuevo León, Monterrey 64460, Mexico; david.paniaguav@uanl.edu.mx (D.P.-V.); orlando.floresmnd@uanl.edu.mx (O.E.F.-M.); aurora.garzajr@uanl.edu.mx (A.d.J.G.-J.)
[3] Secretaría de Ciencia, Humanidades, Tecnología e Innovación (SECIHTI), Mexico City, Mexico; ahuerta@unpa.edu.mx
[4] Universidad del Papaloapan, Instituto de Biotecnología, Centro de Investigaciones Científicas, San Juan Bautista Tuxtepec s/n, Oaxaca 68301, Mexico;
[*] Correspondence: psilva.me0208@uanl.edu.mx; Tel.: +52-(81)-8329-4050
[†] These authors contributed equally to this work.

*$MS^2$ Fragmentation Analysis of Phenolic Acids and Flavonoids Detected in Moringa oleifera Leaf Extract by Direct-Infusion ESI-IT-$MS^2$*

Mass spectrometric analyses were performed by direct flow infusion (DFI) using an LCQ Fleet ion-trap mass spectrometer (Thermo Fisher Scientific®, San Jose, CA, USA) equipped with an electrospray ionization (ESI) source and operated in negative ionization mode (DFI-ESI-IT-$MS^2$). Samples were infused at a flow rate of 15 µL $min^{-1}$. The instrument was operated under the following conditions: capillary temperature, 350 °C; spray voltage, 5.0 kV; capillary voltage, −43 V; and tube lens voltage, −125 V. MS/MS experiments were performed in a data-dependent acquisition mode, selecting precursor ions corresponding to compounds of interest. Collision-induced dissociation (CID) was carried out using collision energies ranging from 20 to 30 eV.

Compound assignment was based on characteristic $MS^2$ fragmentation patterns and v with authentic reference standards, when available, published literature, and publicly available mass spectral databases, including MassBank of North America (MoNA), NORMAN MassBank, and PhytoHub [49-51]. The assignment of chlorogenic acid, kaempferol, quercetin, and rutin was further supported by comparison of their $MS^2$ fragmentation spectra with those of authentic reference standards analyzed under the same experimental conditions. Representative precursor ion and product ion ($MS^2$) spectra of authentic standards and the corresponding ions detected in the *M. oleifera* leaf extract are presented below to support these assignments.

 https://doi.org/10.3390/molecules31162764

**Figure S1. Direct-infusion ESI-IT-MS² analysis of chlorogenic acid**

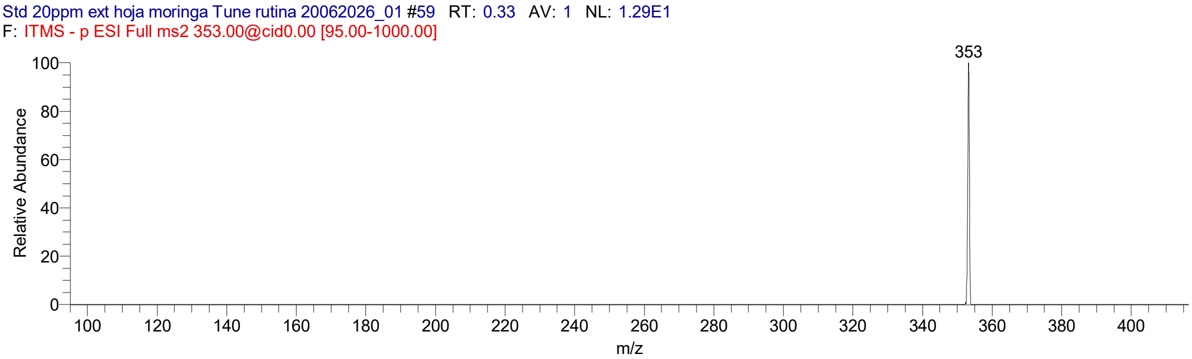


**Figure S1-A**. Precursor ion spectrum of the authentic chlorogenic acid standard showing the deprotonated molecular ion **[M–H]⁻ at m/z 353** (collision energy = 0 eV).

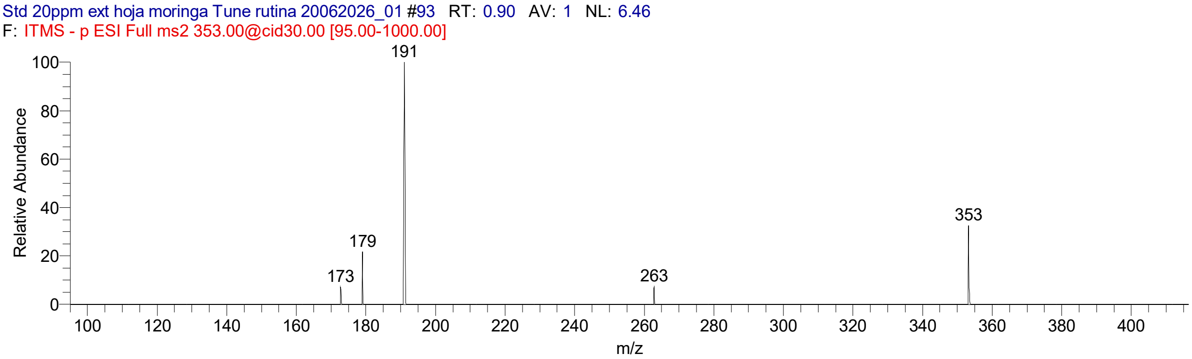


**Figure S1-B**. MS² product ion spectrum of the authentic chlorogenic acid standard acquired from the precursor ion **[M–H]⁻ at m/z 353** (collision energy = 30 eV).

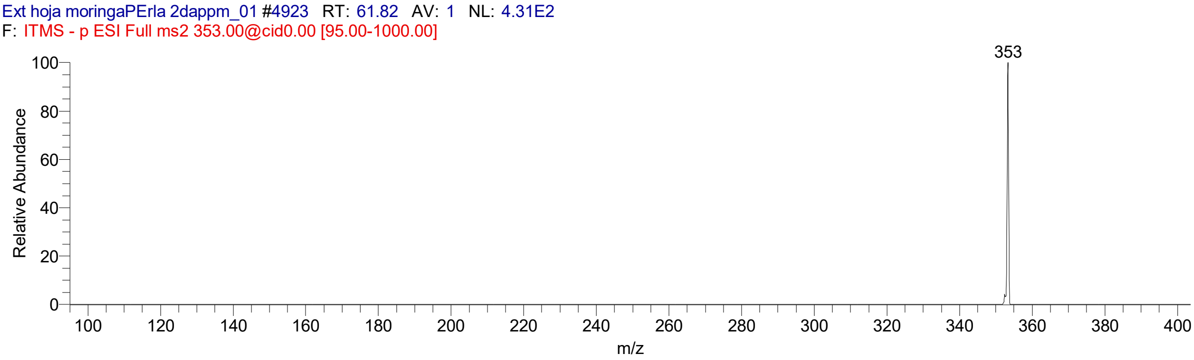


**Figure S1-C**. Precursor ion spectrum of the ion detected in *M. oleifera* leaf extract showing the deprotonated molecular ion **[M–H]⁻ at m/z 353** (collision energy = 0 eV).

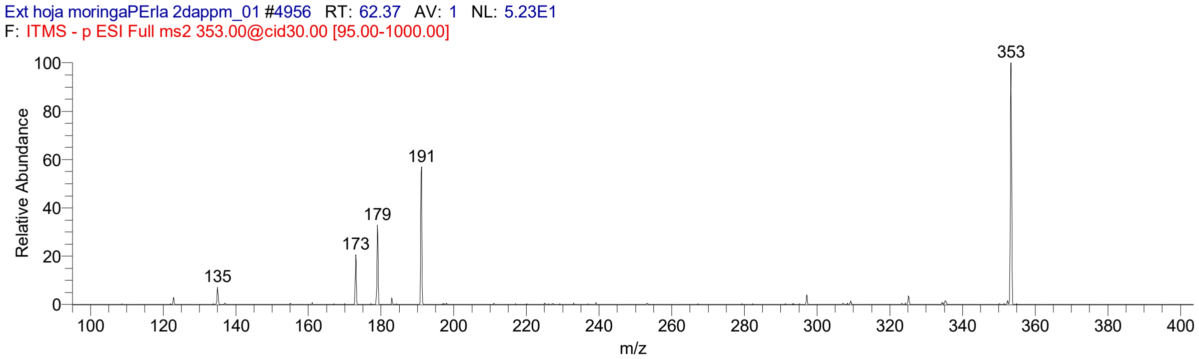


**Figure S1-D**. MS² product ion spectrum of the deprotonated ion **[M–H]⁻ at m/z 353** detected in *M. oleifera* leaf extract and identified as **chlorogenic acid**, showing the characteristic product ions at **m/z 191, 179, 173, and 135** (collision energy = 30 eV).

## Figure S2. Direct-infusion ESI-IT-MS² analysis of kaempferol

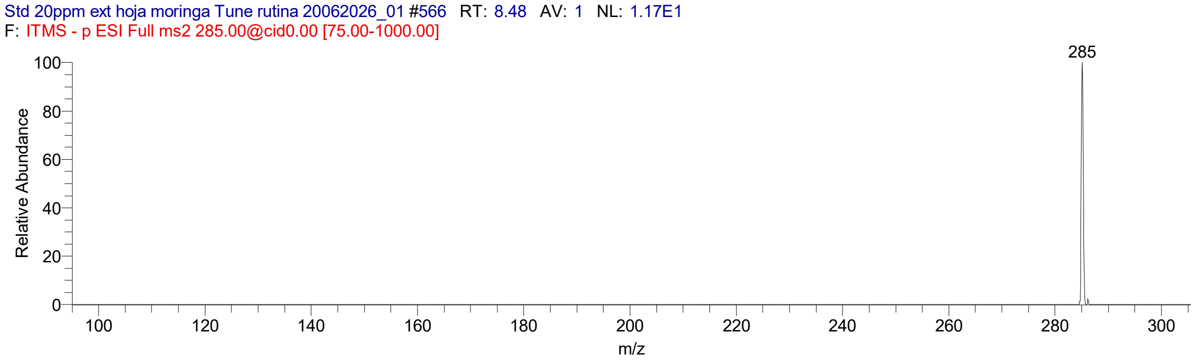


**Figure S2-A**. Precursor ion spectrum of the authentic kaempferol standard showing the deprotonated molecular ion **[M–H]⁻ at m/z 285** (collision energy = 0 eV).

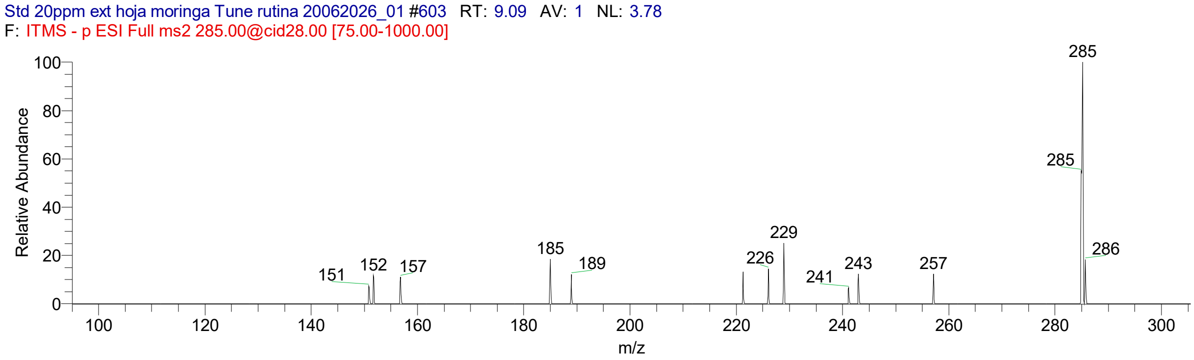


**Figure S2-B**. MS² product ion spectrum of the authentic kaempferol standard acquired from the precursor ion **[M–H]⁻ at m/z 285** (collision energy = 28 eV).

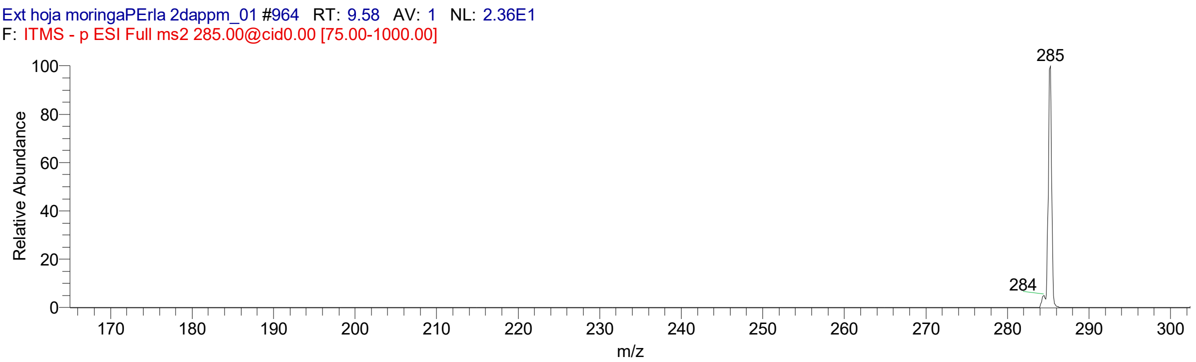


**Figure S2-C**. Precursor ion spectrum of the ion detected in *M. oleifera* leaf extract showing the deprotonated molecular ion **[M−H]⁻ at m/z 285** (collision energy = 0 eV).

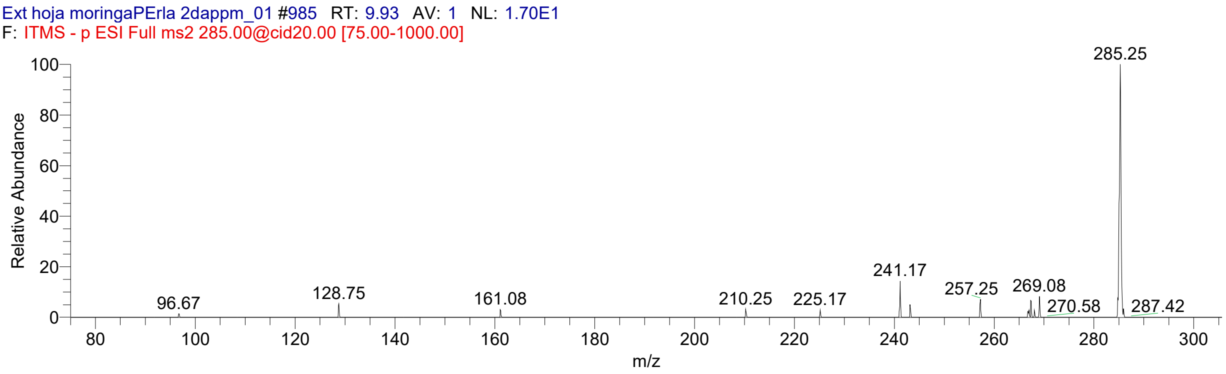


**Figure S2-D**. MS² product ion spectrum of the deprotonated ion **[M−H]⁻ at m/z 285** detected in *M. oleifera* leaf extract and identified as **kaempferol**, showing the characteristic product ions at **m/z 257 and 241** (collision energy = 20 eV).

## Figure S3. Direct-infusion ESI-IT-MS² analysis of quercetin.

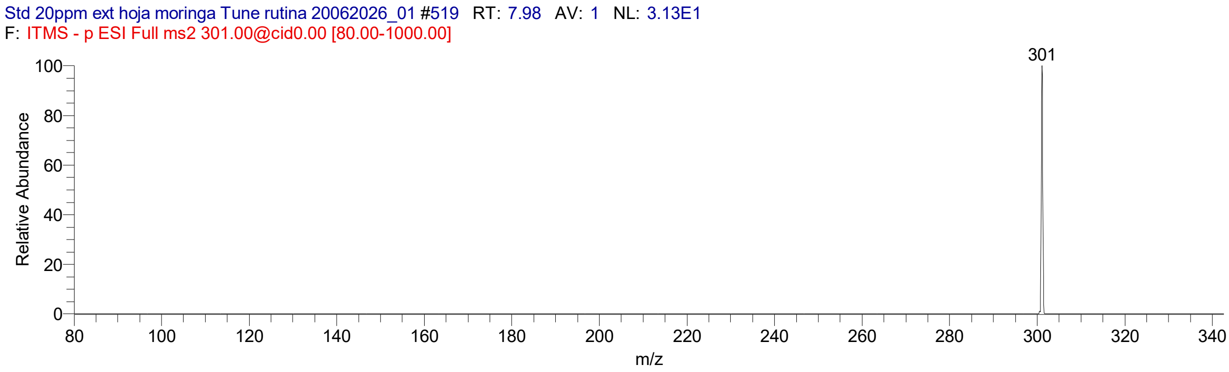


**Figure S3-A**. Precursor ion spectrum of the authentic quercetin standard showing the deprotonated molecular ion **[M−H]⁻ at m/z 301** (collision energy = 0 eV).

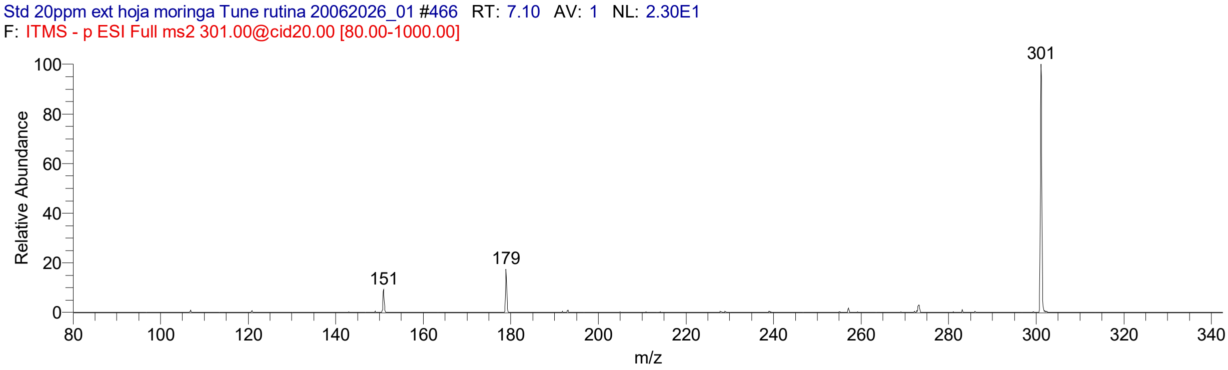


**Figure S3-B**. MS² product ion spectrum of the authentic quercetin standard acquired from the precursor ion **[M−H]⁻ at m/z 301** (collision energy = 20 eV).

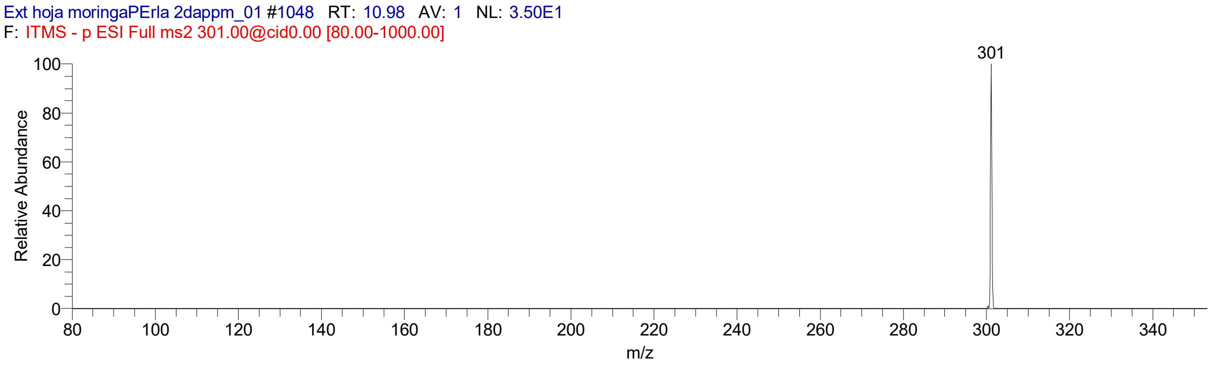


**Figure S3-C**. Precursor ion spectrum of the ion detected in *M. oleifera* leaf extract showing the deprotonated molecular ion **[M−H]⁻ at m/z 301** (collision energy = 0 eV).

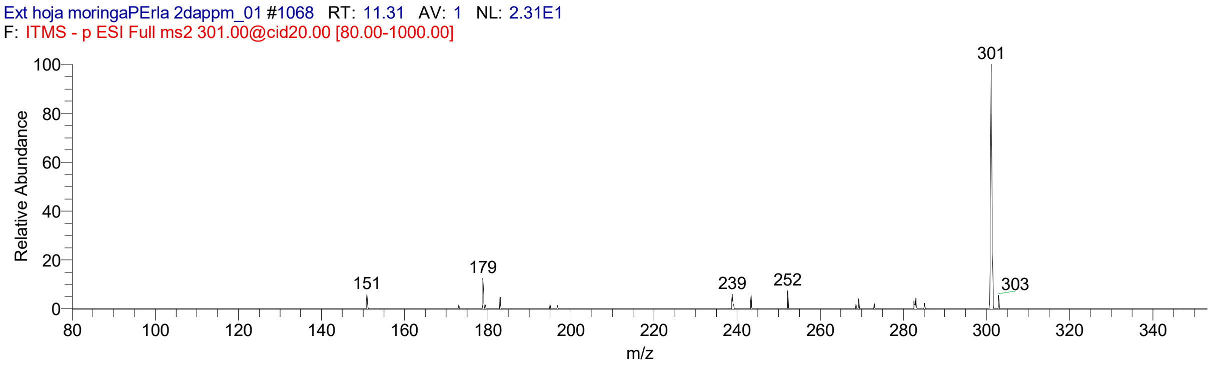


**Figure S3-D**. MS² product ion spectrum of the deprotonated ion **[M−H]⁻ at m/z 301** detected in *M. oleifera* leaf extract and identified as **quercetin**, showing the characteristic product ions at **m/z 179 and 151** (collision energy = 20 eV).

**Figure S4. Direct-infusion ESI-IT-MS² analysis of myricetin**

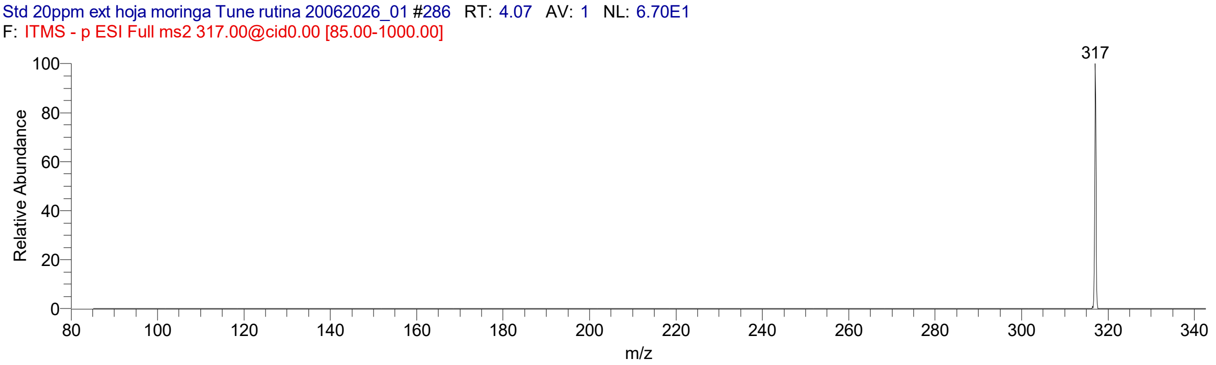


**Figure S4-A**. Precursor ion spectrum of the authentic **myricetin** standard showing the deprotonated molecular ion **[M–H]⁻ at m/z 317** (collision energy = 0 eV).

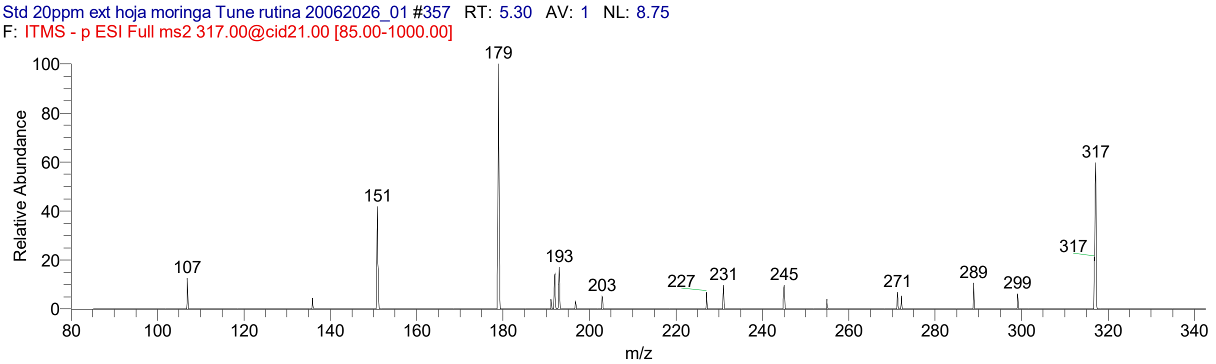


**Figure S4-B**. MS² product ion spectrum of the authentic myricetin standard acquired from the precursor ion **[M–H]⁻ at m/z 317** (collision energy = 21 eV).

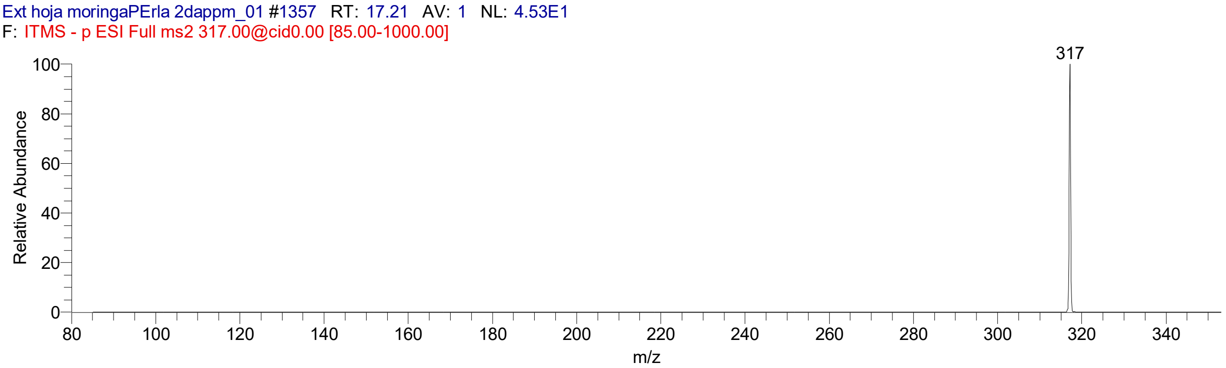


**Figure S4-C**. Precursor ion spectrum of the ion detected in *M. oleifera* leaf extract showing the deprotonated molecular ion **[M–H]⁻ at m/z 317** (collision energy = 0 eV).

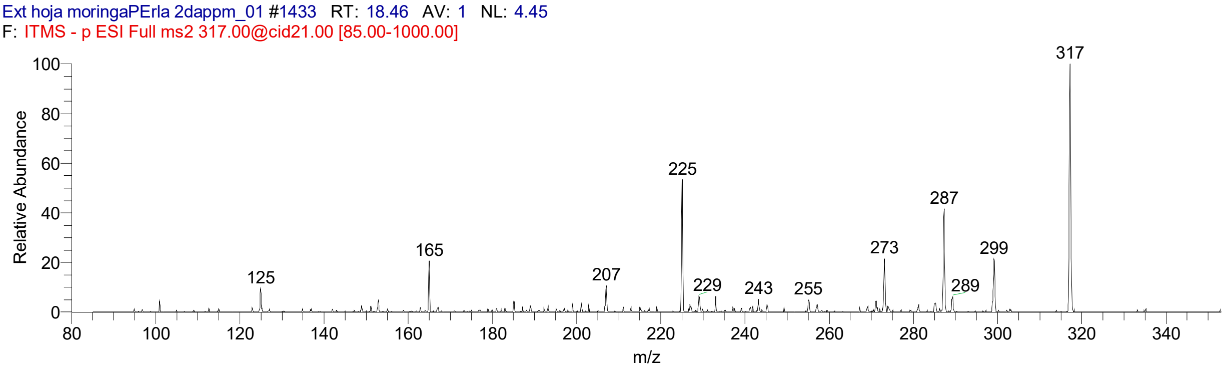


**Figure S4-D**. MS² product ion spectrum of the deprotonated ion **[M−H]⁻ at m/z 317** detected in *M. oleifera* leaf extract and **tentatively assigned as myricetin**, showing the characteristic product ions at m/z 299, 289, and 273.

## Figure S5. Direct-infusion ESI-IT-MS² analysis of rutin

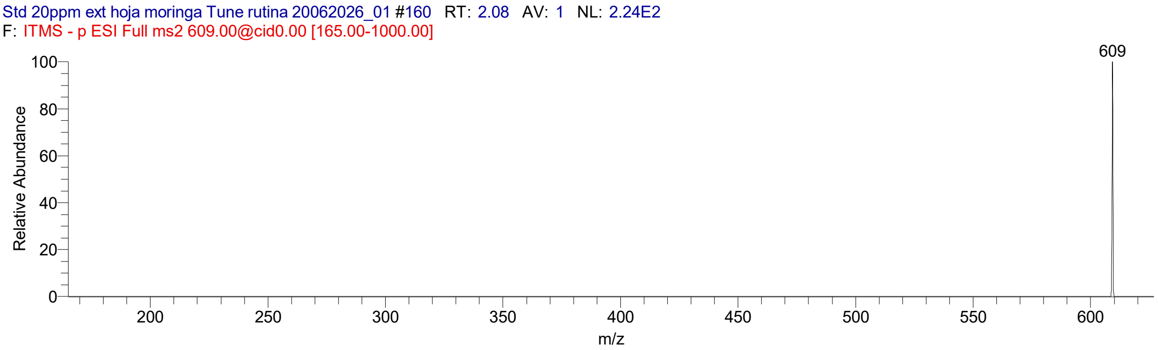


**Figure S5-A**. Precursor ion spectrum of the authentic rutin standard showing the deprotonated molecular ion **[M−H]⁻ at m/z 609** (collision energy = 0 eV).

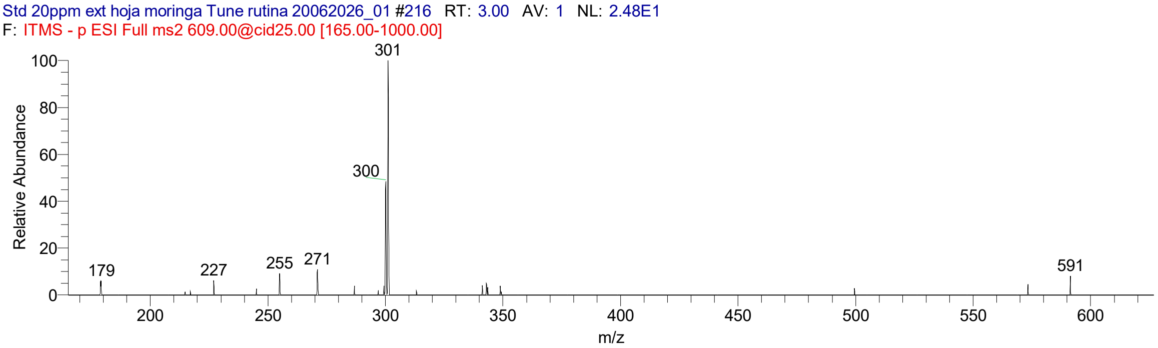


**Figure S5-B**. MS² product ion spectrum of the authentic rutin standard acquired from the precursor ion **[M−H]⁻ at m/z 609** (collision energy of 25 eV).

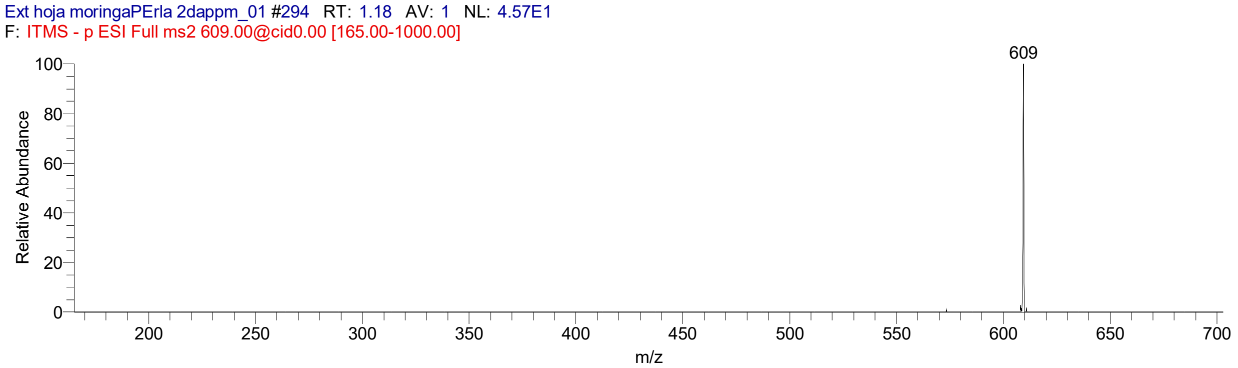


**Figure S5-C**. Precursor ion spectrum of the ion detected in *M. oleifera* leaf extract showing the deprotonated molecular ion **[M–H]⁻ at m/z 609** (collision energy = 0 eV).

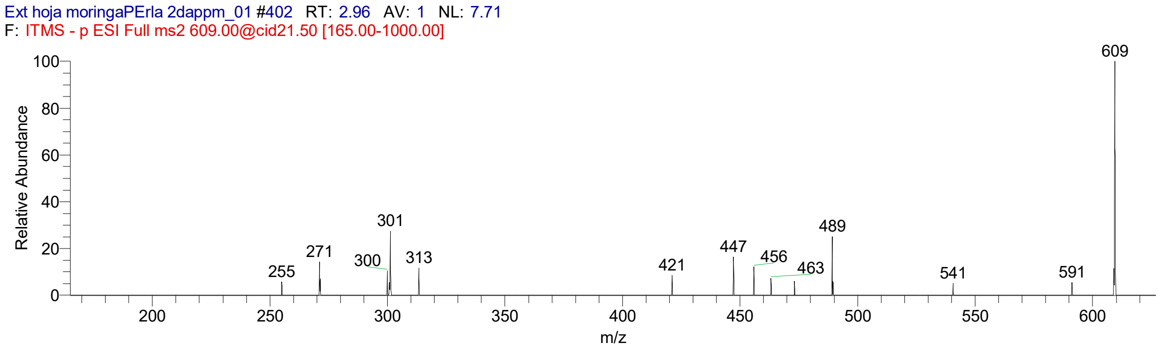


**Figure S5-D**. MS² product ion spectrum of the deprotonated ion **[M–H]⁻ at m/z 609** detected in *M. oleifera* leaf extract and **identified as rutin**, showing the diagnostic product ion **at m/z 301**, corresponding to the quercetin aglycone after the loss of the rutinose moiety, together with secondary product ions at **m/z 300, 271, and 255** (collision energy = 21.5 eV).

## Figure S6. Direct-infusion ESI-IT-MS² analysis of quinic acid

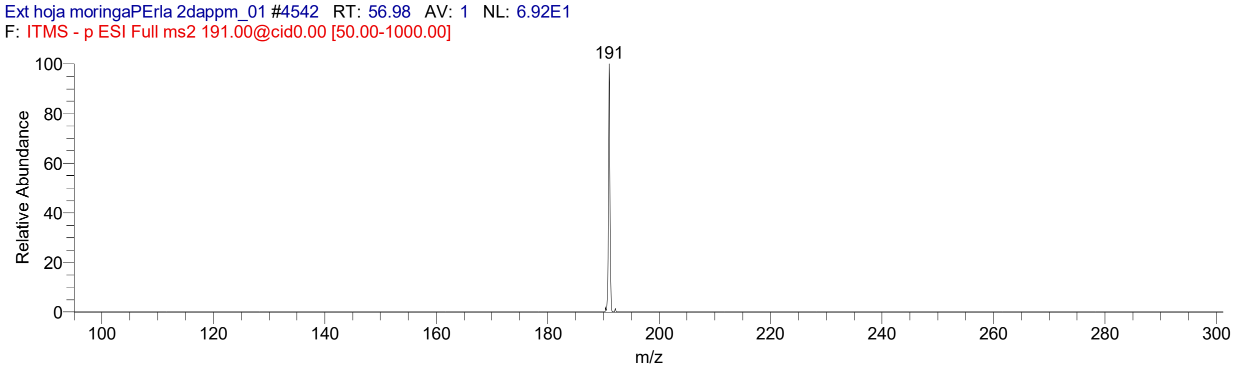


**Figure S6-A**. Precursor ion spectrum of the ion detected in *M. oleifera* leaf extract showing the deprotonated molecular ion **[M–H]⁻ at m/z 191** (collision energy = 0 eV).

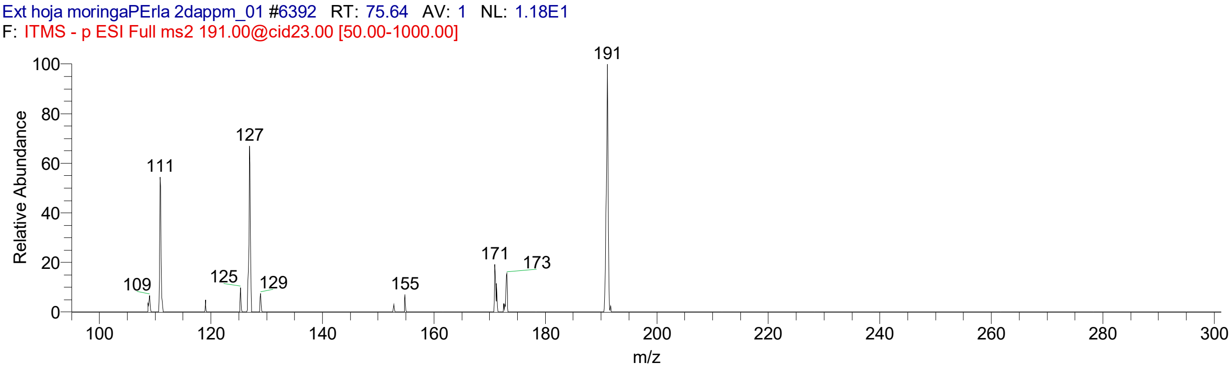


**Figure S6-B**. MS² product ion spectrum of the deprotonated ion **[M–H]⁻ at m/z 191** detected in *M. oleifera* leaf extract and **tentatively assigned as quinic acid** based on its characteristic fragmentation pattern, showing the major product ions at **m/z 173, 127, and 111** (collision energy of 23 eV).

## Figure S7. Direct-infusion ESI-IT-MS² analysis of caffeic acid

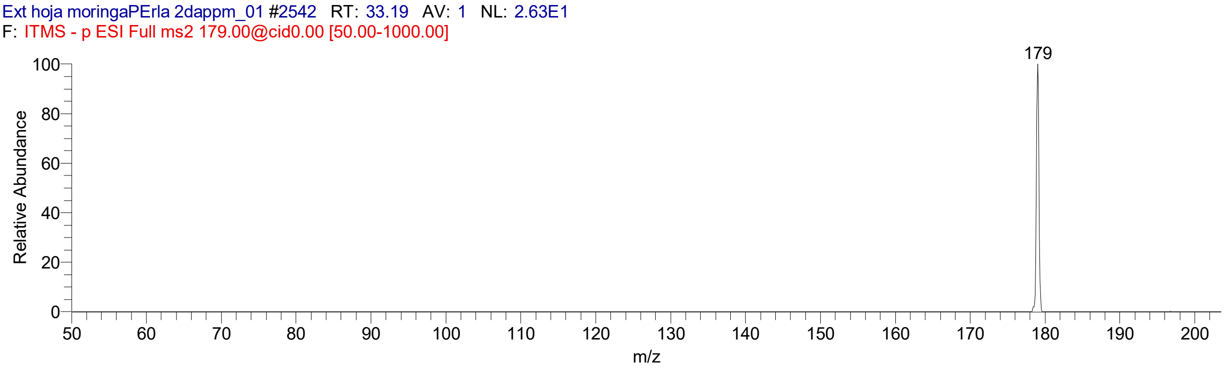


**Figure S7-A**. Precursor ion spectrum of the ion detected in *M. oleifera* leaf extract showing the deprotonated molecular ion **[M–H]⁻ at m/z 179** (collision energy = 0 eV).

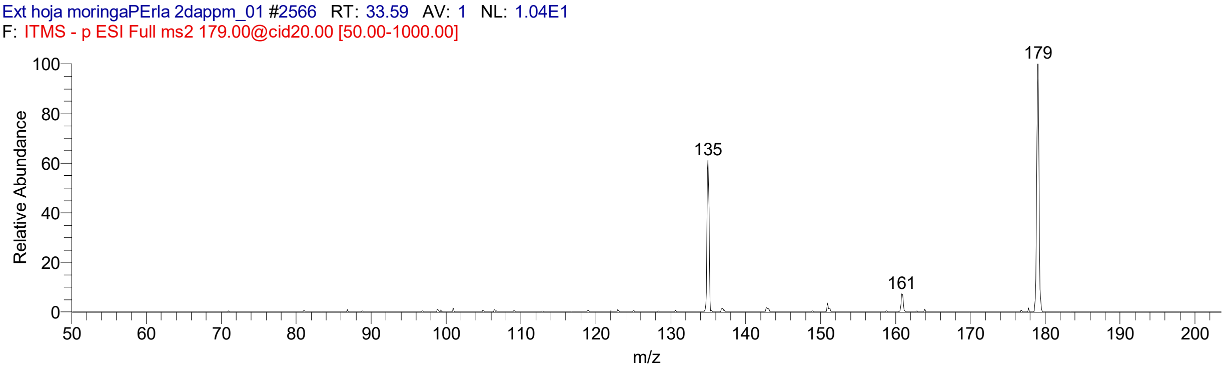


**Figure S7-B**. MS² product ion spectrum of the deprotonated ion **[M–H]⁻ at m/z 179** detected in *M. oleifera* leaf extract and **tentatively assigned as caffeic acid** based on its characteristic fragmentation pattern, showing the major product ions at **m/z 161 and 135** (collision energy of 20 eV).

## Figure S8. Direct-infusion ESI-IT-MS² analysis of kaempferol-O-hexoside

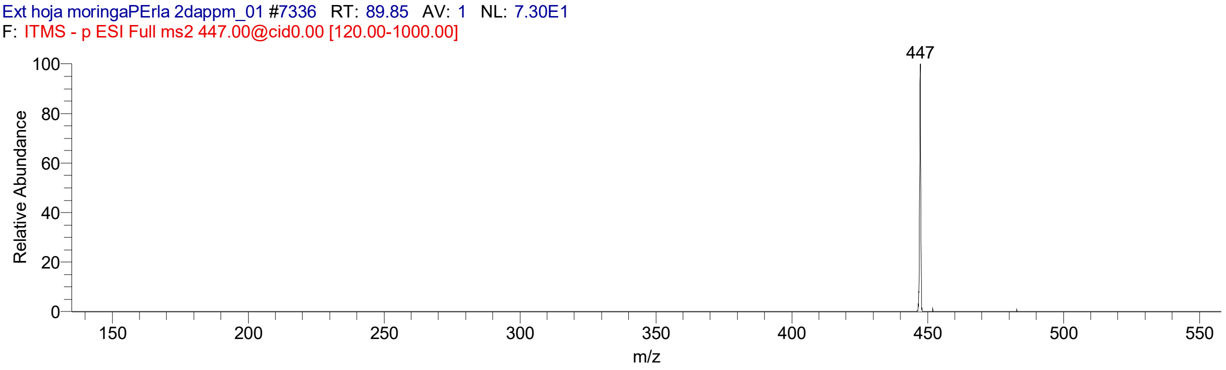


**Figure S8-A**. Precursor ion spectrum of the ion detected in *M. oleifera* leaf extract showing the deprotonated molecular ion **[M–H]⁻ at m/z 447** (collision energy = 0 eV).

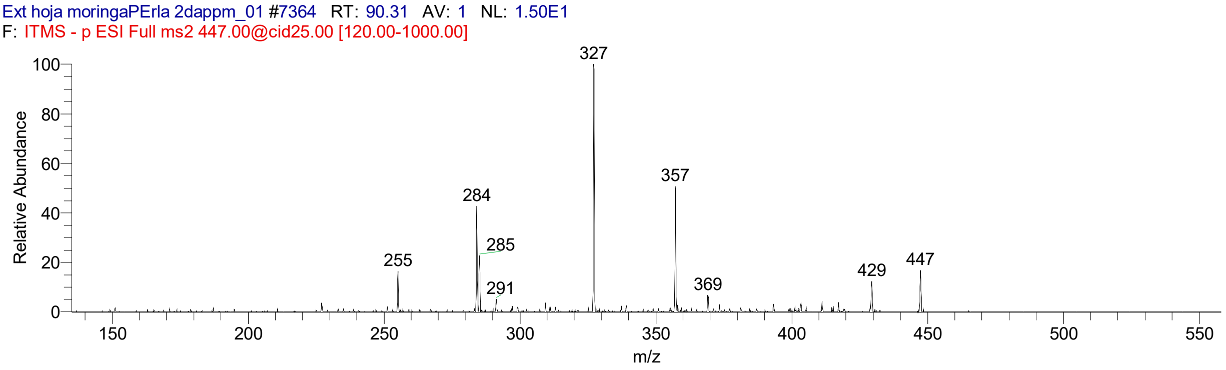


**Figure S8-B**. MS² product ion spectrum of the deprotonated ion **[M–H]⁻ at m/z 447** detected in *M. oleifera* leaf extract and **tentatively assigned as kaempferol-O-hexoside** based on its characteristic fragmentation pattern, showing the diagnostic product ion at **m/z 285**, consistent with the loss of a hexose moiety, together with secondary product ions at **m/z 284 and 255** (collision energy of 25 eV).

## Figure S9. Direct-infusion ESI-IT-MS² analysis of quercetin-O-hexoside

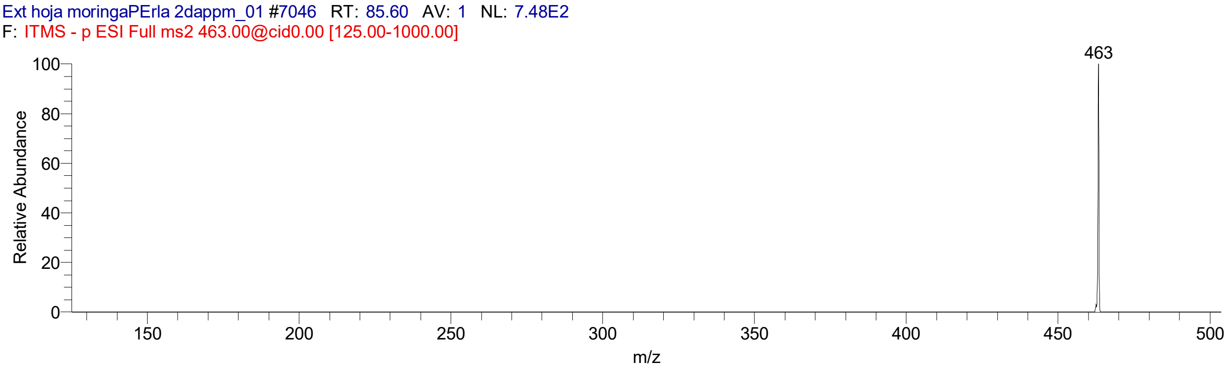


**Figure S9-A**. Precursor ion spectrum of the ion detected in *M. oleifera* leaf extract showing the deprotonated molecular ion **[M–H]⁻ at m/z 463** (collision energy = 0 eV).

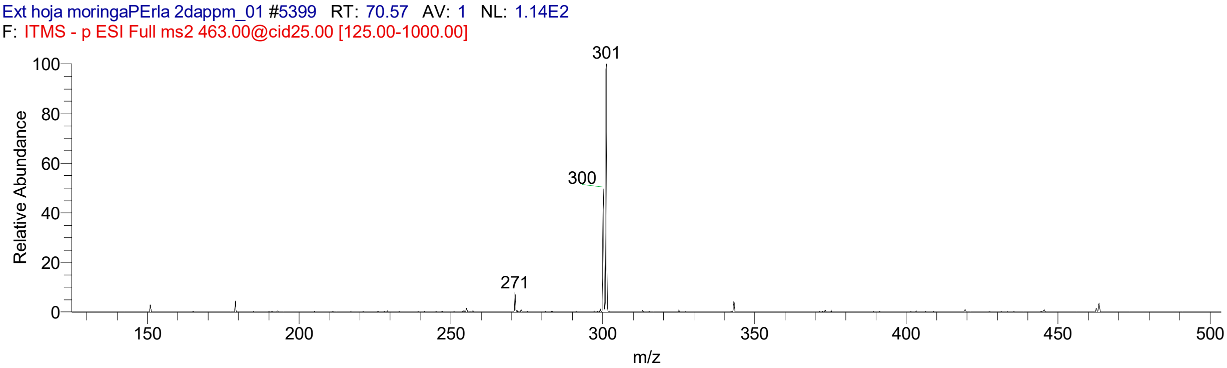


**Figure S9-B**. MS² product ion spectrum of the deprotonated ion **[M−H]⁻ at m/z 463** detected in *M. oleifera* leaf extract and **tentatively assigned as quercetin-O-hexoside** based on its characteristic fragmentation pattern, showing the diagnostic product ion at m/z 301, consistent with the loss of a hexose moiety, together with secondary product ions at **m/z 300 and 271** (collision energy of 25 eV).

## Figure S10. Direct-infusion ESI-IT-MS² analysis of quercetin-O-acetyl-hexose

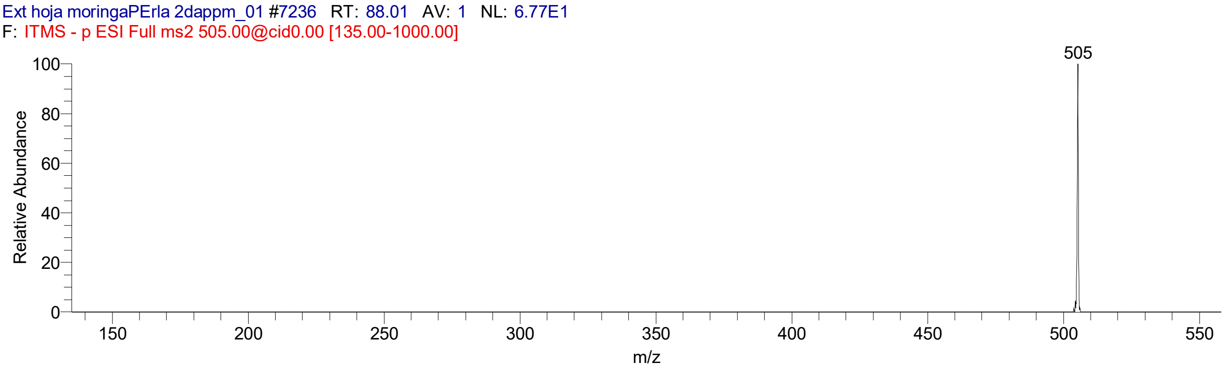


**Figure S10-A**. Precursor ion spectrum of the ion detected in *M. oleifera* leaf extract showing the deprotonated molecular ion **[M−H]⁻ at m/z 505** (collision energy = 0 eV).

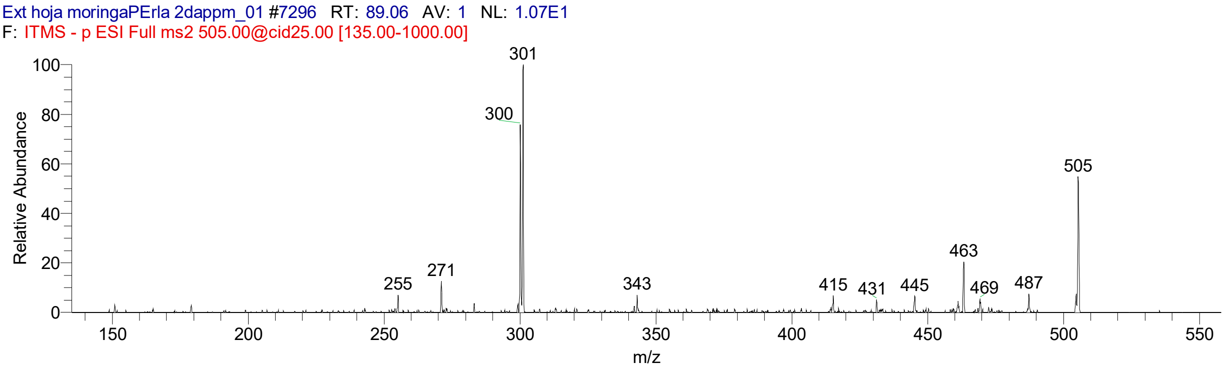


**Figure S10-B**. MS² product ion spectrum of the deprotonated ion **[M−H]⁻ at m/z 505** detected in *M. oleifera* leaf extract and **tentatively assigned as quercetin-O-acetyl-hexose** based on its characteristic fragmentation pattern, showing the diagnostic product ion at **m/z 301**, consistent with sequential losses of an acetyl group and a hexose moiety, together with secondary product ions at **m/z 463, 300, and 271** (collision energy of 25 eV).

**Figure S11. Direct-infusion ESI-IT-MS² analysis of apigenin-C-hexoside**

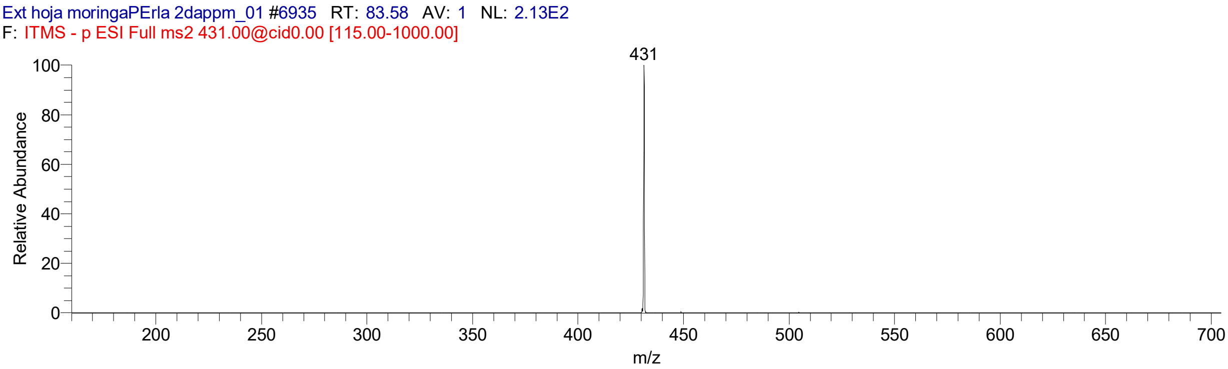


**Figure S11-A**. Precursor ion spectrum of the ion detected in *M. oleifera* leaf extract showing the deprotonated molecular ion **[M−H]⁻ at m/z 431** (collision energy = 0 eV).

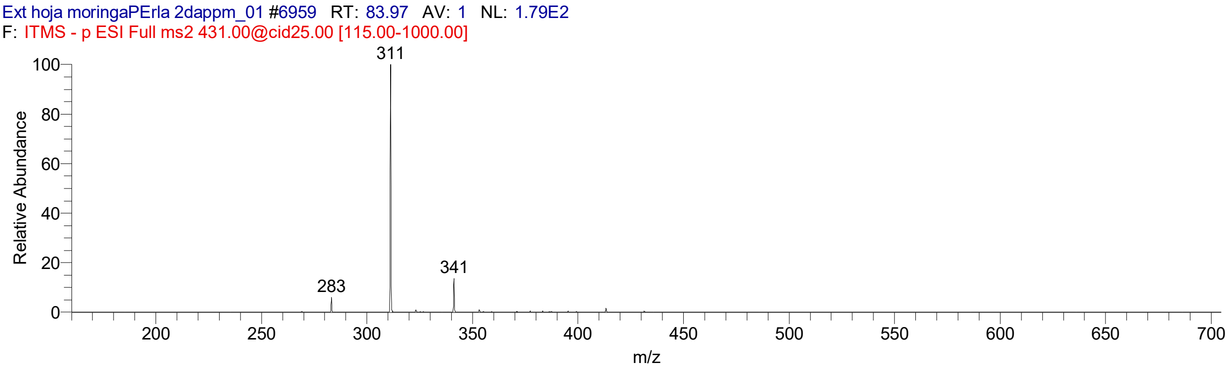


**Figure S11-B**. MS² product ion spectrum of the deprotonated ion **[M−H]⁻ at m/z 431** detected in *M. oleifera* leaf extract and **tentatively assigned as apigenin-C-hexoside** based on its characteristic fragmentation pattern, showing the major product ions at **m/z 341, 311, and 283** (collision energy of 25 eV).
